# Computational Identification of Potential Novel Allosteric IHF Inhibitors Using QSAR Modeling to Inhibit Plasmid-Mediated Antibiotic Resistance

**DOI:** 10.3390/ijms27062526

**Published:** 2026-03-10

**Authors:** Oscar Saurith-Coronell, Olimpo Sierra-Hernandez, Juan David Rodríguez-Macías, José R. Mora, Noel Perez-Perez, Jackson J. Alcázar, Ricardo Olimpio de Moura, Igor José dos Santos Nascimento, Edgar A. Márquez Brazón, Yovani Marrero-Ponce

**Affiliations:** 1 Departamento de Medicina, División Ciencias de la Salud, Universidad del Norte, Km 5, Vía Puerto Colombia, Puerto Colombia 081007, Colombia; osaurith@uninorte.edu.co (O.S.-C.); olimpos@uninorte.edu.co (O.S.-H.); 2 Grupo de Investigaciones en Química y Biología, Departamento de Química y Biología, Facultad de Ciencias Básicas, Universidad del Norte, Carrera 51B, Km 5, Vía Puerto Colombia, Barranquilla 081007, Colombia; 3 Facultad de Ciencias de la Salud, Exactas y Naturales, Universidad Libre, Barranquilla 080001, Colombia; 4 Grupo de Química Computacional y Teórica (QCT-USFQ), Departamento de Ingeniería Química, Universidad San Francisco de Quito, Diego de Robles y Vía Interoceánica, Quito 170901, Ecuador; jrmora@usfq.edu.ec; 5 Colegio de Ciencias e Ingenierías “El Politécnico”, Universidad San Francisco de Quito USFQ, Quito 170157, Ecuador; nperez@usfq.edu.ec; 6 Centro de Química Médica, Facultad de Medicina Clínica Alemana, Universidad del Desarrollo, Santiago 7780272, Chile; jackson.alcazar@udd.cl; 7 Programa de Pós-Graduação em Ciências Farmacêuticas (PPGCF), Universidade Estadual da Paraíba (UEPB), Campina Grande 58429-500, Brazil; ricardo.olimpiodemoura@servidor.uepb.edu.br (R.O.d.M.); igor.n@visitante.uepb.edu.br (I.J.d.S.N.); 8 Facultad de Ingeniería, Universidad Panamericana, Ciudad de México 03920, Mexico; ymarrero@up.edu.mx; 9 Grupo de Medicina Molecular y Traslacional (MeM&T), Colegio de Ciencias de la Salud (COCSA), Escuela de Medicina, Universidad San Francisco de Quito (USFQ), Quito 170157, Ecuador

**Keywords:** antibiotic resistance, plasmid conjugation, Integration Host Factor, QSAR modeling, computational drug design, molecular docking, molecular dynamics

## Abstract

The rapid spread of antibiotic resistance through plasmid-mediated conjugation remains a primary global health concern. Despite its critical role in horizontal gene transfer, no approved drugs currently target this process, leaving a critical therapeutic gap. Integration Host Factor (IHF), a DNA-binding protein essential for plasmid replication and mobilization, emerges as a promising yet underexplored target for anti-conjugation strategies. This work aimed to develop a predictive computational model and identify small molecules that disrupt IHF function, thereby reducing plasmid transfer and limiting resistance gene dissemination. A curated dataset of 65 compounds with reported anti-plasmid activity was analyzed using a 3D-QSAR model based on algebraic descriptors computed with QuBiLS-MIDAS. The model was validated through leave-one-out cross-validation (Q^2^ = 0.82), Tropsha’s criteria, and Y-scrambling. Representative compounds were selected via pharmacophore clustering and evaluated through molecular docking at both the DNA-binding site and a predicted allosteric pocket of IHF. The most promising complexes underwent 200 ns molecular dynamics simulations to assess stability and interaction patterns. The QSAR model demonstrated strong predictive performance (R^2^ = 0.90). Docking simulations revealed more favorable binding energies at the allosteric site (up to −12.15 kcal/mol) compared to the DNA-binding site. Molecular dynamics confirmed the stability of these interactions, with allosteric complexes showing lower RMSD fluctuations and consistent binding energy profiles. Dynamic cross-correlation analysis revealed that allosteric ligand binding induces conformational changes in key catalytic residues, including Pro65, Pro61, and Leu66. These alterations may compromise DNA recognition and disrupt the initiation of replication. To our knowledge, this is the first computational study proposing allosteric inhibition of IHF as an anti-conjugation strategy. These findings provide a foundation for experimental validation and the development of novel agents to prevent horizontal gene transfer, offering a promising approach to restoring antibiotic efficacy against multidrug-resistant pathogens.

## 1. Introduction

Antibiotic resistance has emerged as a critical global public health threat, undermining the effectiveness of antibiotics in treating bacterial infections [1]. Current estimates suggest that bacterial infections are responsible for approximately 7.7 million deaths annually, with nearly 5 million attributed to multidrug-resistant (MDR) pathogens. Infections caused by resistant organisms are not only more challenging to treat but are also associated with markedly prolonged hospital stays. For instance, Gram-negative bacterial infections extend hospitalization by an average of 32.6 days, while severe bloodstream infections can increase hospital stays by up to two months compared with non-infected patients [2]. Beyond the clinical burden, antibiotic resistance imposes substantial economic strain on healthcare worldwide by increasing the consumption of medical resources. Consequently, the World Health Organization has prioritized the development of new therapeutic strategies targeting MDR bacteria [3].

Antimicrobial resistance arises from complex interactions between microorganisms and their environment. In clinical settings, acquired resistance in previously susceptible bacterial populations typically arise from prolonged and uncontrolled antibiotic exposure. Under selective pressure, bacteria develop two principal genetic mechanisms for survival: gene mutation and horizontal gene transfer (HGT) [4,5,6]. Mutations can modify antimicrobial targets, activate enzymatic pathways that inactivate drugs, decrease antibiotic permeability, or induce metabolic changes that bypass drug effects. HGT, recognized as a central driver of antimicrobial resistance emergence and spread, enables bacteria to incorporate foreign genetic material through three distinct pathways: transformation, transduction mediated by bacteriophages, and conjugation, the most common mechanism in clinical settings involving plasmid transfer between cells via direct contact [7,8].

Conjugation is the most important mechanism for horizontal dissemination of antibiotic resistance genes among bacterial populations. This multi-step process begins with cellular contact between donor and recipient cells, mediated by a pilus structure facilitating DNA transfer [9]. The Integration Host Factor (IHF) is a heterodimeric protein (~20 kDa) that binds specifically to DNA and functions as an accessory factor in various cellular processes in prokaryotes, primarily by inducing DNA bending, inducing a U-turn in the DNA structure that is necessary for the assembly of the relaxosome a protein complex. These essential functions position IHF as a promising molecular target for inhibiting plasmid maintenance and horizontal gene transfer, potentially through disruption of its DNA-binding and structural modulation activities [10]. Once cell-to-cell contact is established and plasmid replication is completed, conjugative machinery composed of a type IV coupling protein (T4CP) and type IV secretion system (T4SS) is activated. These genes, typically located on autonomously replicating plasmids, are transcribed upon recognition of the origin of transfer (oriT) by the relaxase protein, which triggers precise cleavage and the mobilization of plasmid DNA [11,12]. Conjugative plasmids are transmitted between bacteria with remarkable efficiency and are classified based on compatibility groups, relaxase families, or the resistance genes they carry. For example, the incompatibility plasmid linage Fertility plasmid group (IncF) belonging to the mobility plasmidum F-type (MOBF) family comprises low-copy number conjugative plasmids (45–200 Kb) commonly found in Gram-negative bacteria, including Escherichia coli, Klebsiella pneumoniae, and Enterobacteriaceae members. Their clinical significance stems from the frequent presence of extended-spectrum β-lactamase genes (ESBLs), such as bla-CTX-M, and carbapenemase genes conferring carbapenem resistance in Enterobacteriaceae (CRE), alongside resistance genes to aminoglycosides, quinolones, and tetracyclines [13,14,15,16,17,18].

While recent antibiotics such as cefiderocol target multidrug-resistant Gram-negative bacteria, this approach offers only short-term solutions. Alternative strategies are being explored, including adjuvants like SPR741, a polymyxin-B–derived cationic peptide that acts as an antibiotic potentiator by disrupting the outer membrane of Gram-negative bacteria, thereby enhancing the efficacy of co-administered antibiotics reducing nephrotoxicity and enabling higher antibiotic dosing [19,20]. However, neither strategy directly addresses the prevention of resistance gene spread or the elimination of the genetic machinery. Currently, no approved clinical drugs directly inhibit horizontal gene transfer in bacteria. Promising compounds under investigation include abacavir and azidothymidine (AZT), antiretroviral analogs reducing pCT plasmid conjugation in *E. coli* by 83% and pKpQIL plasmid conjugation in *K. pneumoniae* by 80%. Linoleic acid, which inhibits TrwD ATPase activity within the T4SS, also demonstrates conjugation-inhibiting properties. These compounds promote plasmid-borne genetic material loss, reducing acquired resistance mechanisms in bacterial populations [21,22].

In silico strategies have emerged as efficient, cost-effective tools for drug development, enabling rapid screening of thousands of potential molecules using computational methods that are significantly faster than traditional approaches. These strategies contribute to a deeper molecular understanding of drug behavior and mechanisms of action, which is crucial for early modifications that optimize biological activity and ADME-Tox properties (absorption, distribution, metabolism, excretion, and toxicity). Molecular docking and molecular dynamics simulations represent the most widely used computational methods, exploring ligand-protein complex conformations and stability while estimating interaction energies. MM-PBSA and MM-GBSA approaches calculate these energies by accounting for bond lengths, angles, torsions, van der Waals interactions, electrostatic forces, and solvent-accessible surface area, enabling optimization of candidate molecules’ pharmacological and toxicological profiles. Quantitative Structure Activity Relationship (QSAR) analysis, a predictive modeling technique increasingly incorporated into drug development, establishes quantitative relationships between compound structural features and biological activity. QSAR models identify molecular characteristics associated with specific pharmacological properties, including mechanism of action, toxicity, and selectivity, enabling informed modifications to optimize performance and safety profiles [23,24,25]. QSAR strategies have been successfully applied to HIV-1 protease inhibitor development, leading to the identification of novel potential inhibitors and the optimization of properties such as hydrophobicity. Clinically available drugs such as Indinavir and Darunavir served as reference compounds guiding the design and refinement of new drugs [26,27,28].

Given the high prevalence of multidrug-resistant organisms that utilize conjugation to transfer virulence and resistance factors, we propose a comprehensive computational approach integrating QSAR modeling, target identification, molecular docking, and molecular dynamics simulations [29,30]. This strategy investigates the mechanisms of action of compounds that demonstrate plasmid conjugation-inhibition activity. Our primary objective is to understand how specific molecules interfere with conjugation, elucidate structure-activity relationships, predict binding affinity and complex stability, and establish a predictive model to guide the development of new potential plasmid conjugation inhibitors. Such inhibitors could meaningfully reduce the spread of antibiotic resistance among clinically relevant bacterial strains.

## 2. Results

To develop a reliable predictive model, we first established a structured methodological sequence. This process began with data collection, followed by the construction of a QSAR model to identify molecular properties that contribute to plasmid conjugation inhibition. Next, a pharmacophore search was conducted to predict potential molecular targets affected by the compounds. Finally, molecular docking and molecular dynamics simulations were performed to analyze interactions between the compounds and their targets, assess the stability of the complexes, and propose plausible mechanisms of action for inhibiting plasmid transfer.

### 2.1. Dataset Preparation

To construct the training dataset for our predictive model, we selected 65 compounds with reported anti-plasmid activity against the pCRE BIDMC20a plasmid. This selection was guided by defined inclusion criteria applied to the ICCB-Longwood Screening Facility database at Harvard Medical School, which houses a chemically diverse array of bioactive molecules, including FDA-approved drugs, kinase inhibitors, ion channel modulators, and other pharmacologically relevant agents [31]. These compounds were chosen to ensure both clinical relevance and mechanistic diversity, providing a solid foundation for subsequent modeling and analysis. All selected compounds were retrieved from the PubChem database https://pubchem.ncbi.nlm.nih.gov (accessed on 22 February 2025), and their corresponding Compound ID (CID) codes were extracted for standardized reference and integration. To facilitate consistent referencing, each compound was assigned a unique identifier in the format ICCB-n. Biological activity was expressed as the Anti-Plasmid Inhibition Index (APII), a metric designed to capture plasmid stability relative to bacterial growth. To calculate APII, we used the ratio of relative fluorescence units (RFU), which reflect plasmid-associated fluorescence, to optical density at 600 nm (OD_600_), an indicator of cell density. This normalization ensures that fluorescence signals are not biased by variations in bacterial growth, providing a more accurate measure of plasmid maintenance under different conditions.

To improve data consistency and reduce skewness, APII values were log-transformed before statistical modeling. Log transformation is a standard practice in high-throughput screening and QSAR studies because it stabilizes variance and helps approximate normality in biological datasets. Similar approaches have been reported in fluorescence-based plasmid transfer assays and conjugation frequency studies [32,33,34,35,36,37]. Therefore, higher log-transformed values correspond to greater inhibition of plasmid maintenance [38]. The complete dataset, including these normalized and transformed values, is presented in Table 1.

Following the initial compound selection, structural refinement was performed using Density Functional Theory (DFT) calculations with the B3LYP functional. To ensure that each optimized geometry represented a true energy minimum, vibrational frequency analyses were performed; only structures exhibiting exclusively positive frequencies were retained [39]. This optimization step enabled the determination of an extensive set of molecular descriptors, encompassing thermodynamic, electronic, and topological properties. In total, approximately 16,000 descriptors were computed for each compound. Subsequent correlation analyses facilitated the development of a predictive mathematical model based on a subset of seven descriptors [40,41]. These selected parameters, primarily algebraic in nature, are presented in Table 2.

In the final model, descriptors X1–X7 correspond to 3D indices generated using the QuBiLS-MIDAS (MID) methodology at the atom-based (AB) level. These descriptors encode geometric patterns between atoms through duplex and ternary relationships, employing different distance metrics and various matrix types, including global indices (T) and, in some cases, full matrix preservation (KA). Regarding their chemical content, X1 is a non-chiral bilinear index associated with a hardness (h) softness (s) term; X2 is a linear, chiral index with kurtosis, using a topological cutoff LGP[2] and weighted by polar surface area (PSA); X3 is a non-chiral bilinear index with skewness that incorporates a terminal-methyl (M) structural term, applies a geometric cutoff LGL[2–3], and is weighted by AlogP (a) and polarizability (p); X4 is a non-chiral bilinear index with skewness, constructed with an LGP[2] cutoff and weighted by electronegativity (e) and polarizability (p); X5 is a linear, chiral index with kurtosis, centered on heteroatoms (X) and weighted by charge (c); X6 is a non-chiral trilinear-cubic index (triplets) including SS2 transformations, cutoffs LG3P[1]/LGP[1], and hardness (h) weighting; and X7 is a linear, chiral index with skewness focused on heteroatoms (X) and weighted by charge (c). Together, these descriptors capture a broad combination of geometric, electronic, and physicochemical information.

The QSAR model points to two complementary factors driving the APII. First, the feature importance analysis based on standardized coefficients and the relative R^2^ contributions shows that descriptors X3, X1, and X2 provide the largest share of explanatory power. All three have negative coefficients, indicating that higher values of the structural and physicochemical patterns captured by X1–X4, particularly polar-surface and electronic-gradient terms, PSA-related information in X2, and the LogP/polarizability/electronic components represented in X3 and X4, are associated with reduced predicted activity. This outcome is consistent with the observation that lower polar surface area, more favorable lipophilicity profiles, and specific electronic-property balances correlate with increased APII within the dataset.

Second, descriptors reflecting charge distribution and electronic stability (X5–X7) exhibit positive coefficients. This suggests a modest beneficial contribution of charge-related patterns (X5 and X7) and global hardness (X6) to activity. However, the importance rankings indicate that these contributions are secondary in magnitude, and only X5 shows strong statistical support in the current model; X6 and X7 contribute positively but more weakly. Taken together, these results indicate that the model preferentially favors ligands with reduced polarity (lower PSA-related signatures) and physicochemical features compatible with membrane penetration and hydrophobic interactions, while charge-distribution patterns and hardness-related stability offer an additional, though comparatively smaller, positive contribution. Overall, this integrated interpretation provides a coherent framework to guide the design of compounds with an improved APII.

### 2.2. QSAR Modeling and Validation

For modeling purposes, the Anti-Plasmid Inhibitor Index (APII) was designated as the dependent variable, and the computed descriptors were used as independent variables. To explore the predictive relationship between molecular structure and biological activity, a range of statistical modeling techniques was employed. Among these, regression-based approaches were particularly instrumental in constructing robust mathematical models capable of correlating the calculated molecular descriptors with the APII [42,43]. Several models were generated during this process; however, one demonstrating the highest statistical significance and predictive performance was selected for further analysis. This final model incorporates the previously described descriptors and is presented in Equation (1).

$$\begin{array}{ll} APII\;=\;0.6445\; & -\;1.5200\;X1\;-\;0.0038X2\;-\;0.0203X3\;-\;0.0363X4\;+\;0.0038X5\;+\;6.0267X6\;\\ \; & +\;0.0046X7 \end{array}$$

N = 65, R^2^ = 0.90, Q^2^ = 0.82 (1)

Analysis of the descriptors included in the model suggests that compounds exhibiting higher biological activity tend to possess greater electronic hardness and branched or cyclic structures. Molecules with a balanced global electronic hardness and softness, combined with intermediate physicochemical properties such as polarizability, charge distribution, logP, and polar surface area (PSA) were favored. The corresponding descriptor values are presented in detail within Supplementary Table S1. Conversely, structures lacking these characteristics were penalized during model optimization. So far, the model indicates a preference for rigid molecular frameworks with regions of high electronic density and a well-defined three-dimensional architecture, as these features appear to enhance the likelihood of effective interaction with the target protein.

**Figure 1:**
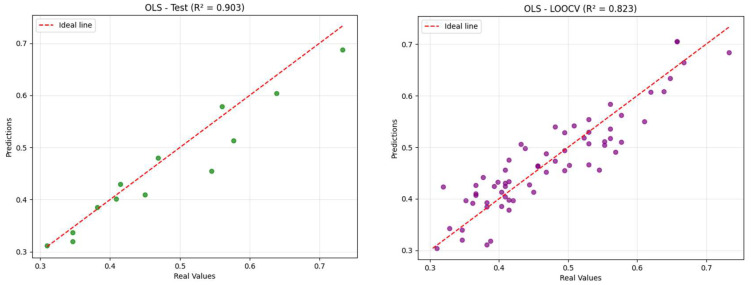
Correlation between the observed APII values and those predicted by the 3D-QSAR model, using both OLS regression and LOOCV approaches.

To validate the predictive performance of the model, leave-one-out cross-validation (LOOCV) was employed [44]. This technique involves iteratively excluding one compound from the dataset, training the model on the remaining *n* − 1 compounds, and then predicting the excluded compound’s activity. The process is repeated for each compound, and the average prediction error across all iterations is calculated to assess model reliability [45]. The resulting model demonstrated strong predictive power, with a cross-validated coefficient of determination (Q^2^) of 0.8232, alongside low error metrics (RMSEP = 0.0413; MAE_ext_ = 0.0337). Figure 1 compares predictions obtained using the OLS-Hold-Out method and LOOCV.

To ensure that all training molecules fell within the applicability domain of the QSAR model, a Williams plot was generated (Supplementary Figure S1). This plot shows standardized residuals reflecting prediction accuracy against leverage values, which indicate each compound’s influence on the regression model. Higher leverage values correspond to a stronger impact on the equation. The horizontal boundaries at ±3σ (three standard deviation units) define the acceptable residual range, while the vertical threshold at h = 0.369 represents the calculated leverage limit. All training compounds were located within these boundaries, confirming the absence of structural or response outliers. This outcome supports the reliability of the predictions and reinforces the robustness and interpretability of the QSAR model [46,47,48].

Additional validation of the model was carried out using Tropsha’s criteria for predictive QSAR models. In Table 3, K and K′ represent the slopes of the regression lines obtained from the plot of predicted versus observed activities for the external and internal validation sets, respectively; values close to 1 indicate that the QSAR model has appropriate scaling and is neither over- nor under-estimating the response. Moreover, the value of ∣R^2^ − R_0_^2^∣/R^2^ was 0.0354, reflecting strong model robustness. Similarly, the difference ∣R_0_^2^ − R_0_′^2^∣ was below 0.1, further supporting the reliability of the model [49,50]. A comprehensive summary of the statistical analysis and metric validation is presented in Table 3.

To further assess the model’s robustness, a Y-scrambling analysis was performed. This validation technique yielded negative R^2^ values (mean = −0.19; standard deviation = 0.10), effectively ruling out spurious correlations and confirming the statistical validity of the model. Additionally, the model’s performance was compared with alternative regression algorithms. Notably, the Random Forest approach produced a lower predictive accuracy (R^2^ = 0.55), underscoring the superior performance of the linear model. These findings support the appropriateness of the linear structure in capturing. All those validation schemes support the predictive reliability of the model to predict the APII of different molecules [51]. Taken together, these validation approaches support the model’s reliability in predicting APII values across structurally diverse compounds. However, it is important to recognize that the relatively small number of compounds used during the development of the QSAR model may limit its performance when applied to external datasets. This reduced dataset size could increase the risk of overfitting and, consequently, hinder the model’s ability to generalize to new chemical structures [52].

### 2.3. Cluster Analysis and Selection of Representative Compound

Following the development of the predictive model, a cluster analysis was conducted to identify structurally related groups among the 65 compounds used in model training. The three-dimensional structures of these molecules were utilized to generate pharmacophore maps using the pmapper Python 3.12.3 library, which integrates the feature-detection capabilities of RDKit https://www.rdkit.org (accessed on 1 April 2025). Key pharmacophoric features such as hydrogen bond donors and acceptors, hydrophobic regions, aromatic rings, and ionizable groups were included.

**Figure 2:**
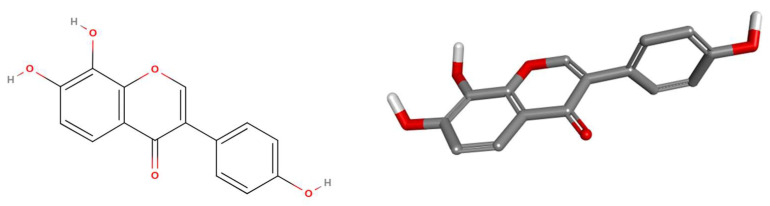
3D structure of the representative compound, ICCB-3.

Each feature was encoded based on its 3D coordinates, establishing a geometric center that reflects the principal structural characteristics of each molecule. These pharmacophores were then represented using canonical signatures and 2048-bit binary fingerprints. Importantly, the 3D signatures allowed for the identification of pharmacophoric similarities across compounds without requiring molecular alignment, thereby avoiding biases introduced by differences in molecular size. To ensure consistency, spatial parameters such as distances and planarity were discretized using tolerance settings defined by pmapper. The resulting fingerprints were used to compute Tanimoto similarity scores, which served as the basis for clustering [53]. This process yielded a major cluster comprising 31 compounds, from which a single representative molecule was selected. The remaining compounds were distributed across smaller clusters. The representative compound of the largest cluster is ICCB-3 (8-Hydroxydaidzein), illustrated in Figure 2.

ICCB-3 has a molecular weight of 270.24 g/mol and belongs to the isoflavone, a class of natural compounds within the flavonoid family, a group of polyphenols predominantly found in legumes. Isoflavones have garnered significant interest in pharmaceutical research due to their antioxidant and cardiovascular properties, as well as their potential role in hormone-dependent cancers [54,55].

### 2.4. Target Prediction

To explore the potential mechanism of action underlying the observed inhibitory activity, a target prediction analysis was conducted using PharmMapper https://www.lilab-ecust.cn/pharmmapper (accessed on 5 April 2025), to identify plausible biological targets for the representative compound ICCB-3. Its 3D structure was first used to generate pharmacophoric features [56,57]. These features were then aligned and analyzed using PharmMapper. To enhance the accuracy and flexibility of the mapping process, a conformer search was performed, generating approximately 500 conformations using the MMFF94 force field. The pharmacophore mapping was conducted against a curated database of 16,159 druggable pharmacophore models. This extensive dataset includes experimentally validated protein-ligand interactions derived from natural ligands, known drug targets, and repurposed drugs. Based on pharmacophoric similarity, PharmMapper identified approximately 1000 potential targets. From this pool, a rigorous selection process was undertaken to prioritize targets involved in key biological processes such as plasmid replication, regulation, and transfer. Among the top candidates, two targets were identified, with particular emphasis on the Integration Host Factor (IHF).

### 2.5. Docking Protocol

Following the QSAR analysis and subsequent target identification, we proceeded with molecular docking simulations to deepen our understanding of the potential mechanisms of action of the selected compounds. This approach enabled us to explore potential interactions between the ligands and IHF, providing valuable insights into their binding behavior. By integrating QSAR modeling with molecular docking techniques, we aimed to establish a qualitative and, potentially, quantitative relationship between molecular structure and biological activity [58,59]. The docking simulations were conducted using IHF as the target protein, focusing on a set of 31 compounds that belong to a representative cluster. These molecules were selected based on their apparent shared mechanism of action, which involves plasmid inhibition. The combined computational strategy not only supports the hypothesis of a typical interaction pathway but also enhances the predictive power of our analysis regarding ligand efficacy and specificity [60,61].

The initial hypothesis proposes that the selected compounds interfere with the interaction between IHF and plasmid DNA. This inhibition could occur through two primary mechanisms: (i) direct binding to amino acid residues involved in DNA recognition, or (ii) induction of conformational changes in the protein that alter its interaction with DNA, potentially through an allosteric mechanism [62]. To investigate these possibilities, two distinct approaches were used to define the docking grid parameters. The first approach relied on a comprehensive literature review to identify key residues responsible for DNA binding. Among these, Pro65 was highlighted for its role in binding to the minor groove and inducing DNA bending. Additional residues, such as Arg63 and Thr60, were also considered for their functional importance in IHF-DNA interactions [63]. Based on this information, the docking grid was centered at X: 2, Y: 14, Z: 4, with dimensions of 50 Å × 50 Å × 50 Å. The second approach involved a cavity search using the web-based platform CBDock2 and the open-source tool fpocket. CBDock2 integrates three methodologies: solvent-accessible surface area (SASA) analysis, structure-based docking with AutoDock Vina 1.1.2, and template-based docking, which compares the target protein with structurally similar proteins and their known ligands [64]. In parallel, fpocket employs a Voronoi tessellation algorithm to partition space into regions of atomic influence, identifying empty pockets on the protein surface and interior that may serve as druggable sites. Both methods converged on the same potential allosteric pocket, which was selected as the second docking grid, centered at X: −2, Y: −9, Z: 34, with the same dimensions of 50 Å × 50 Å × 50 Å [65].

Molecular docking simulations were performed using AutoDockGPU, a high-performance tool that leverages GPU resources to efficiently generate a large number of potential binding poses between the receptor and each ligand [66]. For each compound, 1000 conformations were generated, using both docking grids previously described: one targeting the DNA-binding site and the other a potential allosteric site. The resulting docking data were analyzed based on several parameters: the best binding energy (lowest conformational energy), the mean binding energy across all poses, the standard deviation of the energy values, and the root mean square deviation (RMSD) of the conformational clusters formed between the ligand and the active site. These metrics provided a comprehensive view of the binding stability and variability for each compound. The results of the docking simulations are summarized in Table 4, which presents data for both proposed mechanisms of action: direct interaction with DNA-binding residues and allosteric modulation of the IHF structure.

**Figure 3:**
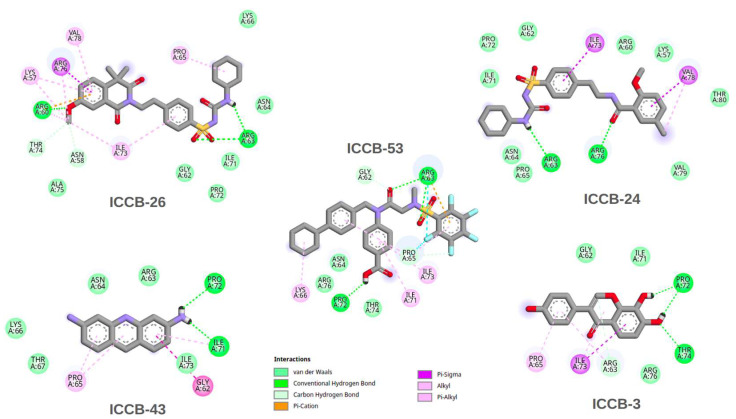
2D interaction map illustrating the key molecular contacts observed in the docking results for the most representative compounds bound to the IHF protein via the direct inhibition mechanism.

**Figure 4:**
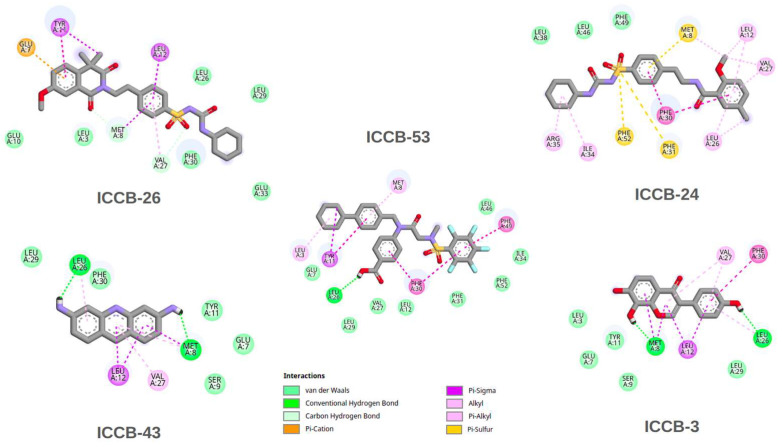
2D interaction map illustrating the key molecular contacts observed in the docking results for the most representative compounds bound to the IHF protein via the allosteric inhibition mechanism.

The results of the molecular docking simulations provide valuable insights into potential interactions between the selected compounds and the IHF protein. Moreover, correlating these findings with the QSAR model outcomes contributes to validating the predictive power of the computational approach. In general, the binding energies associated with the direct inhibition mechanism ranged between −6 and −9 kcal/mol. In contrast, the docking results targeting the proposed allosteric site showed slightly more favorable binding energies, ranging from −7 to −11 kcal/mol. This suggests that the compounds may interact more effectively with the allosteric region of the protein. Among the tested molecules, ICCB-26 stood out for the most favorable binding energy at the allosteric site and for strong interaction in the direct inhibition model. Notably, this compound also demonstrated the highest biological activity in experimental assays. ICCB-24 showed high binding affinity and significant biological activity, whereas ICCB-53, despite its strong binding energy, displayed lower biological activity. This apparent discrepancy between docking scores and biological activity suggests that additional factors may influence the observed bioactivity. These could include differences in membrane permeability, compound stability, or the possibility of a multitarget mechanism of action. To further elucidate these findings, a detailed analysis of the molecular interactions between the compounds and IHF was conducted, focusing on both the DNA-binding site and the proposed allosteric site. To further explore the molecular basis underlying the docking and biological activity results, a detailed interaction analysis was performed for ICCB-26, ICCB-24, and ICCB-53, as well as ICCB-3 the representative compound of the cluster and ICCB-43, which exhibited the weakest performance in both docking simulations and biological assays. The chemical structures of these compounds are provided in Supplementary Figure S2. The interactions of the docking results for the analysis of direct inhibition are shown in Figure 3, and those for allosteric inhibition are shown in Figure 4.

The molecular interaction analysis derived from the docking simulations revealed a diverse range of non-covalent interactions between the ligands and the IHF protein; These included Van der Waals forces, conventional hydrogen bonds, carbon-hydrogen bonds, π-cation, π-sigma, alkyl, and π-alkyl interactions. Notably, within the allosteric pocket, several complexes display π-sulfur (π-S) contacts, wherein the ligand’s aromatic π-system interacts favourably with the polarizable sulfur of methionine/cysteine, contributing to pose stabilization and orientational bias. This interaction is well documented in proteins: methionine-aromatic motifs are common and provide ~1–1.5 kcal·mol^−1^ of additional stabilization, and sulfur-involving noncovalent contacts are increasingly recognized as determinants of protein structure and function; in line with this, proximity to aromatic residues has been shown to reduce methionine’s susceptibility to oxidation [67,68].

As illustrated in Figure 3, ICCB-26 exhibited a high number of π-sigma, π-alkyl, and hydrogen bond interactions, along with van der Waals contacts. These interaction types were also present in ICCB-53, supporting the hypothesis that such interactions may contribute to the favorable docking scores observed for these compounds. In contrast, ICCB-24 and ICCB-43 displayed fewer of these key interactions, which may explain their comparatively lower binding affinities. ICCB-3, used as the reference molecule, helped identify the most relevant residues involved in ligand binding. Among these, Pro 65 stood out for its critical role in DNA binding and in inducing the characteristic U-turn in the DNA structure. Other vital residues included Arginine 76, Arginine 63, and Isoleucine 73. The compounds interacted with these residues through different mechanisms, including hydrogen bonding, π-sigma, and alkyl interactions, highlighting the structural diversity in ligand-receptor engagement.

**Figure 5:**
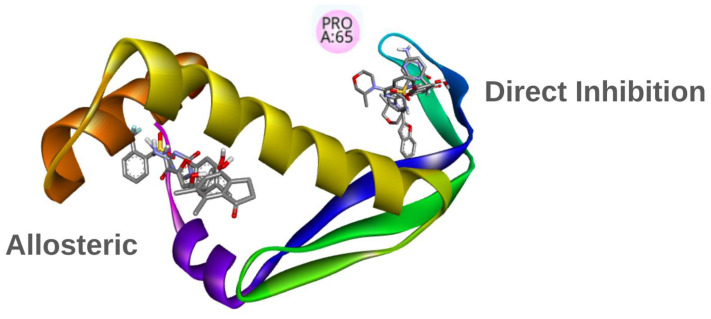
Validation of the redocking protocol for assessing both direct and allosteric inhibition mechanisms targeting the IHF protein.

In contrast to the results from direct inhibition docking simulations, Figure 4 shows that compounds ICCB-26 and ICCB-24 exhibit distinct interaction profiles. These molecules interact by Pi-Sulfur and Pi-Cation interactions, alongside conventional hydrogen bonds and Van der Waals forces. Such interactions are likely to contribute significantly to the enhanced binding scores observed during docking, suggesting a potential role in stabilizing the ligand-protein complex. Conversely, ICCB-53, despite demonstrating a favorable binding score, showed poor biological activity. Notably, this compound lacks Pi-Sulfur and Pi-Cation interactions, which may account for its reduced efficacy. Although ICCB-53 forms Pi-Sigma, Alkyl, van der Waals, and hydrogen bond interactions, the absence of Pi-Sulfur contacts appears to be a critical factor influencing its functional performance. Similarly, ICCB-43 lacks these key interactions and exhibits fewer overall molecular contacts, further supporting this hypothesis. Among the compounds analyzed, ICCB-3 serves as a representative example, exhibiting consistent and meaningful interactions with key amino acid residues, including Leu 12, Leu 26, and Phe 30. These residues participate in hydrogen bonding and Pi-Sigma interactions, which may play a pivotal role in the compound’s mechanism of action. If the inhibitory effect of these molecules is mediated through this interaction pathway, their interaction with these residues could induce conformational changes that disrupt DNA binding, alter protein folding, or interfere with dimerization processes essential for the protein’s active structure. These possibilities warrant further investigation through molecular dynamics simulations, which could provide deeper insights into the conformational behavior of the protein-ligand complexes. Figure 5 illustrates the results of the redocking validation procedure performed with AutoDock Vina (version 1.2.7), showing the superposition of all compounds within both the allosteric and direct inhibition pockets of the IHF protein [69,70]. The visualization highlights their spatial arrangement and key interactions with relevant residues, including Pro65.

This comparative analysis was designed to elucidate specific interaction patterns and binding behaviors at both the DNA-binding site and the proposed allosteric site of the IHF protein. By examining the nature and strength of interactions such as hydrogen bonds, hydrophobic contacts, and electrostatic forces across these regions, we aimed to identify structural determinants that influence the inhibitory potential of the compounds. The inclusion of ICCB-3 as a reference compound provided a consistent framework for evaluating interaction profiles within the cluster, while ICCB-43 served as a contrasting example, highlighting structural and chemical features associated with reduced activity. All ligands analyzed share an extended polyaromatic scaffold with peripheral carbonyl and/or hydroxyl substituents, forming a rigid π-conjugated core that enables π–π and π–alkyl interactions within the hydrophobic pocket, complemented by polar groups capable of establishing directional hydrogen bonds with key residues. ICCB-3, in particular, represents the most compact and symmetric analogue, resembling a substituted anthraquinone in which oxygenated groups are directly attached to the tricyclic aromatic core without bulky sulfonyl or amide side chains. This architecture minimizes rotatable bonds and heteroatom diversity, yielding a highly planar, conformationally restricted scaffold whose interactions are dominated by centrally located hydrogen bonds and extensive aromatic and hydrophobic contacts. These observations support the hypothesis that biological efficacy depends not only on binding energy but also on the quality and nature of molecular interactions within the target site [71].

### 2.6. Molecular Dynamics Simulations

**Figure 6:**
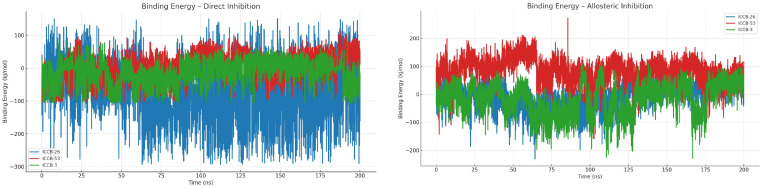
Variation in binding energy (expressed in kJ/mol) throughout a 200-nanosecond molecular dynamics simulation for both direct and allosteric inhibition mechanisms of the IHF protein.

To gain deeper insight into the potential mechanisms of action of the plasmid inhibitors and into how these compounds form stable complexes with their targets, molecular dynamics (MD) simulations were conducted to assess the conformational stability and binding energy of the ligand-protein complexes over time. The compounds ICCB-26 and ICCB-53 were selected for this analysis due to their strong performance in docking simulations and their contrasting biological activity profiles, which represent two extremes in the experimental results. This contrast is particularly valuable for exploring possible correlations between in silico predictions and in vitro efficacy. Additionally, ICCB-3 was included as a reference compound due to its representative interaction profile. Its inclusion may help define the minimum interaction requirements necessary to elicit a biological response. Following the docking protocol, two MD simulations were performed for each compound, corresponding to the two proposed mechanisms of action. The initial conformations used for the simulations were derived from the lowest energy docking poses. Simulations were carried out using YASARA Structure, employing the AMBER14 force field, which has been previously validated for modeling interactions between proteins and small molecules [72,73,74]. Each simulation was run for 200 nanoseconds, allowing sufficient time to observe dynamic behavior and interaction stability. For the initial analysis, two key parameters were evaluated: the binding energy (Figure 5) and Root Mean Square Deviation (RMSD) (Figure 6). These metrics provide insight into the binding affinity and structural stability of the complexes throughout the simulation period, offering valuable information on the potential effectiveness and reliability of the compounds as inhibitors [75].

Throughout the molecular dynamics simulations, distinct interaction behaviors were observed between the compounds and the target protein, depending on the inhibitory mechanism being evaluated. Binding energy was calculated using the YASARA macro md_Analyzebindenergy.mcr, which estimates this metric as the difference between the solvation and potential energies of the ligand-protein complex and those of the individual reactants. This analysis employs the MM-PBSA (Molecular Mechanics Poisson–Boltzmann Surface Area) methodology, integrating an entropy approximation to yield a more comprehensive binding energy profile. Within this framework, the calculated binding energy values are interpreted such that more positive values indicate stronger and more favorable interactions, whereas more negative values correspond to weaker or unfavorable interactions [76]. The results revealed that allosteric inhibition generally produced more favorable and stable interaction energies. For instance, the reference compound ICCB-3 exhibited a mean interaction energy of −15.10 kJ/mol, while ICCB-26 and ICCB-53 showed values of −6.53 kJ/mol and 84.39 kJ/mol, respectively. On the other hand, the direct inhibition mechanism yielded less favorable and more variable energy profiles. ICCB-3 recorded a mean interaction energy of −12.27 kJ/mol, whereas ICCB-26 and ICCB-53 showed values of −64.22 kJ/mol and 5.11 kJ/mol, respectively. A clear trend emerged from these results: ICCB-53 consistently showed the most favorable energy interactions across both inhibition mechanisms, suggesting a strong and stable binding affinity. On the other hand, ICCB-26 displayed highly variable interaction energies, suggesting not only a weaker binding affinity but also instability in its interaction with the target over time.

To complement and deepen the interpretation of the binding energy analysis, it is essential to consider the RMSD of the entire protein-ligand complex. The combined evaluation of these two metrics provides a more comprehensive understanding of the interaction dynamics and stability of the system throughout the simulation. In the case of allosteric inhibition, the RMSD values indicate a relatively stable interaction across all compounds, with fluctuations averaging around 3 Å. Notably, a stabilization period was observed during the first 20 nanoseconds, likely reflecting initial conformational adjustments as the system reached an energetically favorable state following structural optimization. Conversely, the direct inhibition mechanism exhibited greater conformational variability, with RMSD values reaching approximately 4 Å. The compound ICCB-53 showed the highest instability, undergoing fluctuations of up to 6 Å during the first 75 nanoseconds before settling into a more stable conformation. This behavior suggests that the system required a longer period to reach equilibrium, possibly due to initial dynamic instability [77].

**Figure 7:**
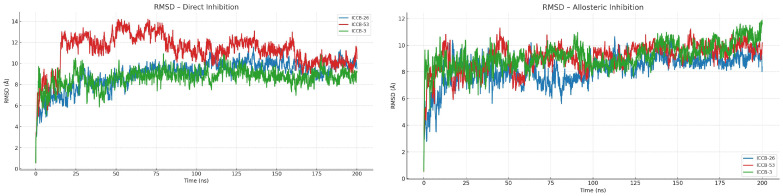
RMSD profiles over the simulation time for both direct and allosteric inhibition mechanisms of the IHF protein.

Taken together, the binding energy and RMSD analyses suggest that the allosteric inhibition mechanism is associated with stronger and more stable interactions, supporting the hypothesis that it may represent the primary mode of action by which these compounds inhibit plasmid transfer. However, to fully understand how this mechanism operates, it is crucial to examine the specific interactions between the ligands and the protein, and how these interactions influence the protein’s structural behavior. These molecular interactions between the compounds and the IHF protein are illustrated in Figure 7, providing visual insight into the nature and location of the binding contacts.

**Figure 8:**
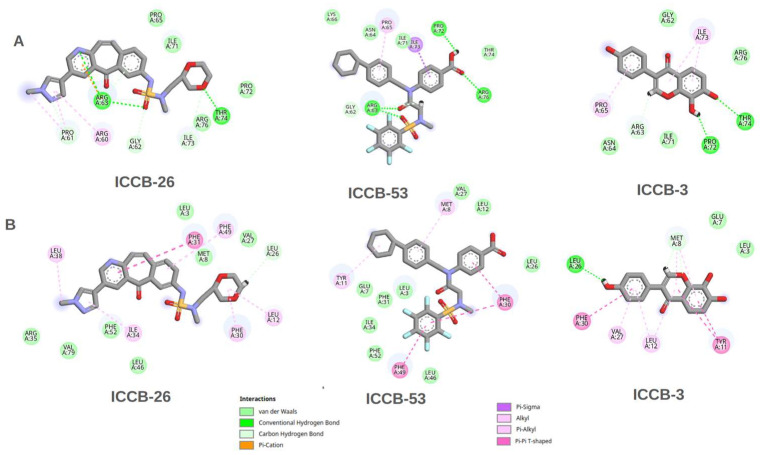
2D interaction map illustrating the key molecular contacts observed in the MD results for the most representative compounds bound to the IHF protein via the direct (**A**) and allosteric (**B**) inhibition mechanisms.

As shown in Figure 8, in the direct inhibition scenario, a high number of classical hydrogen bonds were observed, particularly involving residues such as Arg63, Thr74, and Pro72. Although these interactions occur within the catalytic site and are generally strong, they appear to be highly sensitive to minor conformational changes in either the protein or the ligand. This sensitivity is reflected in the binding energy and RMSD analyses, which suggest a less stable interaction over time. In contrast, the allosteric site is characterized by a hydrophobic and aromatic pocket, with frequent non-polar contacts between the ligands and residues such as Phe30, Phe31, Tyr11, and Met8. The most prominent interactions in this region are of the types of Pi–Pi stacking and Pi–Alkyl, which likely play a crucial role in maintaining the stability of the complex throughout the simulation. Moreover, these interactions may contribute to conformational changes that indirectly affect the active site, particularly residues like Arg63 and Pro65.

**Figure 9:**
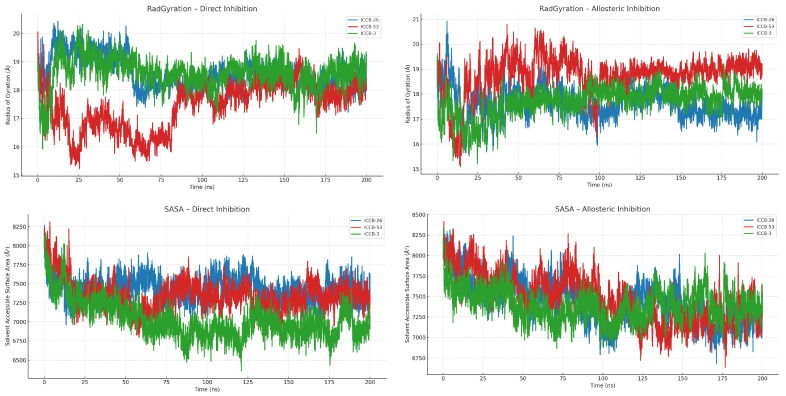
Temporal evolution of the Rg and SASA throughout the molecular dynamics simulations for both direct and allosteric inhibition mechanisms of the IHF protein.

To further investigate these structural modifications, we analyzed the radius of gyration (Rg) and the solvent-accessible surface area (SASA), as presented in Figure 9. These metrics provide additional insight into the compactness and exposure of the protein structure, helping to clarify the impact of ligand binding on overall protein dynamics [78].

The analysis of the Rg and SASA provides valuable analysis into the overall conformational changes in the protein during molecular dynamics simulations. These metrics are particularly useful for assessing both the degree of structural compaction and the distribution of solvent-exposed regions, which may reflect alterations in the protein’s tertiary structure [79]. Notable differences were observed between the two inhibitory mechanisms. In the case of direct inhibition, the protein exhibited a greater degree of compaction, as indicated by a lower average radius of gyration. This observation was further supported by reduced SASA, suggesting that the protein adopts a more compact conformation upon ligand binding at the catalytic site. In contrast, the allosteric inhibition mechanism was associated with a larger solvent-accessible surface area, implying a more relaxed or expanded conformation. This may result from conformational changes in distal secondary structure elements, potentially induced by ligand binding at the allosteric site.

**Figure 10:**
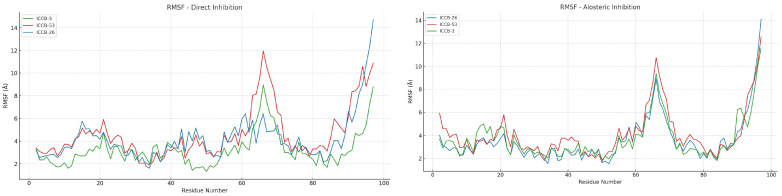
RMSF profiles obtained from molecular dynamics simulations for both direct and allosteric inhibition mechanisms of the IHF protein.

To further investigate which residues undergo the most significant structural fluctuations, and to understand how these changes might influence the protein’s functional activity, we analyzed the Root Mean Square Fluctuation (RMSF) of each residue. These results are presented in Figure 10, providing a residue-level view of the protein’s dynamic behavior under different inhibitory conditions.

**Figure 11:**
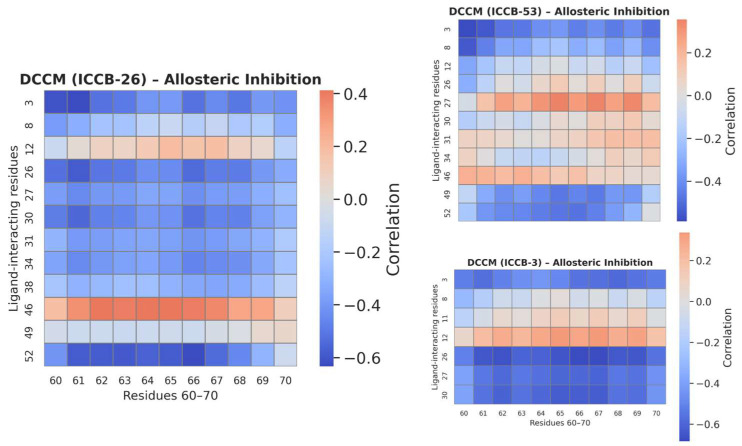
DCCM analysis, highlighting the correlated motions between the ligand and the contact residues within the IHF protein, particularly those associated with the DNA-binding site.

The RMSF analysis revealed a consistent pattern of conformational changes across both proposed mechanisms of inhibition. In both simulation sets, the most significant fluctuations were observed in the region encompassing residues 60 to 70, notably affecting Pro65, a residue previously identified as functionally relevant. These findings suggest that this region may play a central role in the structural response of the protein to ligand binding. In the case of allosteric inhibition, the observed fluctuations were more consistent across the different compounds, supporting the hypothesis of a shared mechanism of action among molecules belonging to the same conformational cluster identified within the applicability domain of the QSAR model [80]. This consistency may indicate a common structural pathway through which these compounds exert their inhibitory effects. On the other hand, the direct inhibition mechanism also showed fluctuations in the same residue region; however, the pattern was less uniform across the compounds. For instance, ICCB-26 did not exhibit the same degree of structural variation observed in other compounds, suggesting a less stable or less consistent interaction with the protein. Additional fluctuations in other regions of the protein further support the notion of a less stable conformational state under direct inhibition. Of particular interest is the observation that, despite binding at a site distant from the active center, compounds acting through allosteric inhibition were still able to induce structural changes in key catalytic residues such as Arg63 and Pro65. To explore how these distal interactions might influence the active site, a Dynamic Cross-Correlation Matrix (DCCM) analysis was performed (Figure 11). This approach allows for the identification of correlated motions between residues interacting with the ligands and those located in the active site, offering further insight into the allosteric communication pathways within the protein.

The DCCM analysis between the residues involved in ligand binding and those located in the active site of the protein provides valuable insight into the mechanistic relationship between ligand interaction and functional modulation. This analysis helps to explain the observed correlation between biological activity and the results of the in silico simulations. In the DCCM plots, blue regions represent anti-correlated motions, where residues move in opposite directions, while red regions indicate positively correlated motions, meaning the residues move in the same direction [81,82]. White areas reflect a lack of correlation between residue movements. The compound ICCB-26, which exhibited the highest biological activity, showed strong anti-correlated motions between residues in the active site, including Pro65 and those interacting directly with the ligand. This suggests a possible mechanical decoupling between the binding site and the catalytic core, potentially mediated by conformational changes in the β-sheets and loops that form the active site. Such decoupling could hinder the protein’s ability to interact effectively with DNA, thereby inhibiting its function. This behaviour has been reported in several works in which an allosteric mechanism is suggested [83,84].

In contrast, ICCB-53, which demonstrated the lowest biological activity, displayed positively correlated motions between the ligand-binding residues and the active site. This may indicate a lack of structural disruption, allowing the protein to maintain its functional conformation despite ligand binding. Notably, the most significant anti-correlated interactions in the ICCB-26 complex were observed between Phe52–Pro65, Leu26–Pro61, and Leu26–Lys66. These residues may represent key nodes within the allosteric pocket, whose dynamic behavior has a pronounced impact on the structural integrity of the active site. For ICCB-3, which exhibited intermediate biological activity, a mixed pattern of correlated and anti-correlated motions was observed.

This observations aligns with the moderate efficacy observed and suggests that partial allosteric modulation may be occurring. All compounds interact with a common set of hydrophobic hotspots LeuA3/A26, ValA27, PheA30/31, and TyrA11 but differ significantly in how these contacts are distributed along the binding cleft. ICCB-26 and ICCB-53 establish extended, multi-point interaction networks: distal aromatic rings engage peripheral Leu and Phe residues, while the central sulfonyl group forms persistent polar contacts with residues such as GluA7 and IleA34, effectively bridging adjacent subsites within the pocket. In contrast, ICCB-3 concentrates its interactions around PheA30, TyrA11, and LeuA26, with a shorter interaction radius and fewer peripheral van der Waals contacts. This pattern reflects a more localized anchoring within the central cavity and limited engagement of lateral subpockets. Such an interaction topology supports ICCB-3 as a minimal binder that retains essential pharmacophoric contacts but lacks the extended subpocket coverage observed for ICCB-26 and ICCB-53. These findings underscore the importance of long-range dynamic interactions in modulating protein function and reinforce the hypothesis that allosteric inhibition may represent a more effective strategy for disrupting IHF activity [85].

## 3. Discussion

Antibiotic resistance is a major global health threat that compromises the effectiveness of antimicrobial therapies. It occurs when bacteria develop mechanisms to evade antibiotics, often through complex interactions with their environment. Resistance can arise through horizontal gene transfer, in which bacteria acquire resistance genes from other microorganisms, or through selective pressure from prolonged antibiotic exposure, especially in aquatic environments. Additionally, genetic mutations in susceptible bacteria can alter drug targets or enzymatic pathways, reducing antibiotic binding and leading to treatment failure [86,87]. Bacterial conjugation is a key mechanism for the spread of antibiotic resistance. This process uses a sex pilus to transfer plasmids between cells. Among these, IncF plasmids are particularly important because they carry genes for resistance to drugs such as β-lactams, carbapenems, and tetracyclines.

This study implemented an integrated computational approach combining 3D-QSAR predictive modeling, pharmacophore-based screening, molecular docking, and molecular dynamics simulations to predict and propose the potential biological effects of compounds with demonstrated plasmid inhibition activity. The first step involved developing a predictive QSAR model using a dataset of 65 compounds and seven molecular algebraic descriptors that capture electronic, thermodynamic, and topological properties, calculated with QuBiLS-MIDAS. The resulting model exhibited high predictive performance (R^2^ = 0.90, Q^2^ = 0.82) and was validated through multiple strategies, including Leave-One-Out Cross-Validation (LOOCV), Tropsha’s criteria, and Y-scrambling, confirming its robustness and reliability. Analysis of the selected descriptors revealed a preference for rigid molecular frameworks with regions of high electronic density and a well-defined three-dimensional architecture. Furthermore, the presence of a balanced distribution of electronic hardness and softness, along with intermediate physicochemical properties such as polarizability, charge, logP, and polar surface area (PSA), was associated with enhanced interaction with the target protein and improved biological activity.

Following the QSAR analysis, a pharmacophore-based search was conducted to identify representative compounds from the original set of 65 molecules used to build the predictive model [88]. This process involved generating pharmacophoric clusters based on molecular fingerprints calculated using pmapper and analyzed with RDKit. Key features such as hydrogen bond donors and acceptors, hydrophobic regions, aromatic rings, and ionizable groups were considered to create a 3D pharmacophore representation of all compounds in the dataset. From these clusters, a central compound was selected to represent the largest group based on structural similarity and biological activity. As a result, ICCB-3 was chosen as the representative compound. This molecule was subsequently used for target identification through the PharmMapper web platform, which performs extensive searches for potential druggable pockets using a large database of receptor-ligand complexes. The analysis identified the Integration Host Factor (IHF) as the most promising target among more than 1000 candidates, a conclusion supported by bibliographic evidence. IHF plays a critical role in plasmid replication, which is essential for bacterial conjugation. Multiple studies have shown that structural alterations in this protein can significantly impair its normal function, leading to a reduction in plasmid transfer efficiency. These structural changes disrupt the interactions between the monomers that form the functional dimer, resulting in improper dimerization of the complex. In addition, modifications in key residues involved in DNA recognition interfere with the initiation of the plasmid-transfer process. Together, these findings highlight the importance of the protein’s structural integrity and demonstrate that even subtle modifications can directly compromise plasmid replication [89,90]. Structurally, it is a heterodimer with a central α-helical core flanked by β-sheets that mediate DNA binding. Proline residues, such as Pro65, interact with DNA bases, inducing bends in the double helix that facilitate origin activation, DNA unwinding, and assembly of replication proteins, including RepE and DnaA. Additionally, IHF interacts with components of the type IV secretion system (T4SS), such as TraY and Tram, promoting DNA curvature required for plasmid mobilization. This coordinated activity accelerates the dissemination of antibiotic resistance genes. Therefore, inhibiting IHF function represents a promising strategy to block plasmid replication and limit the spread of antimicrobial resistance [91,92].

The Integration Host Factor (IHF) was selected as the molecular target for docking simulations, evaluating two potential inhibition mechanisms: direct inhibition of the active site where the protein interacts with DNA to induce structural modifications and allosteric inhibition, which triggers conformational changes that alter protein functionality. Overall, the allosteric mechanism exhibited more favorable binding energies. Notably, ICCB-26 achieved strong docking scores in both mechanisms (>8.6 kcal/mol) and displayed the highest Anti-Plasmid Inhibition Index (APII). In contrast, ICCB-53 also showed good binding energies (>8.4 kcal/mol) but poor biological activity, suggesting that APII is influenced not only by ligand-protein affinity but also by additional factors such as compound permeability and complex stability over time.

The molecular dynamics (MD) simulations confirmed both the affinity and stability of the ligand-protein complexes. Simulations were performed over 200 nanoseconds, providing strong evidence in favor of an allosteric inhibition mechanism, as compounds bound to this site exhibited greater stability than those that interacted directly. The mean RMSD for these complexes was approximately 3 Å, and binding energy values reached up to 84.4 kJ/mol, indicating a highly stable interaction. Additional analyses, including radius of gyration, solvent-accessible surface area (SASA), and root mean square fluctuation (RMSF), offered further insights into the structural dynamics of the protein-ligand complexes. These metrics revealed conformational changes and stability patterns that reinforce the hypothesis of a potential allosteric mechanism of inhibition [93].

Despite these favorable results, a direct correlation with biological activity has not yet been fully established. To address this, and considering the findings from the RMSF analysis, which revealed positional changes in active-site residues, particularly Pro65 and Arg63, under the allosteric mechanism, we examined the interactions of the compounds at their minimum-energy conformations during the simulation. This analysis identified key residues involved in complex formation at the allosteric site, including Phe30, Phe31, Tyr11, and Met8, which primarily engaged in Pi–Pi, Pi–Alkyl, and van der Waals interactions. To further explore the functional impact of these interactions, a Dynamic Cross-Correlation Matrix (DCCM) was generated to assess the relationship between residues in the allosteric pocket and those in the active site. The results revealed a clear correlation with biological activity: compounds with higher activity tended to induce anti-correlated motions between interacting and functional residues, suggesting that this dynamic decoupling may enhance inhibitory effects.

These anti-correlated motions may be associated with a mechanical decoupling between the allosteric binding site and the catalytic core, likely mediated by conformational changes in the β-sheets and loops that form the active site. Such structural rearrangements could impair the protein’s ability to interact effectively with DNA, thereby inhibiting its biological function [94]. Although this mechanism may provide a plausible explanation for the observed changes in biological activity, it is also important to address the apparent decoupling between binding stability and the experimental activity reported. This discrepancy suggests that the mechanism of action proposed could play a decisive role. Typically, molecules that inhibit a protein by binding directly to the active site show a relationship between their binding stability and the corresponding biological activity. However, in the present case, an allosteric mechanism has been proposed. Under such circumstances, the primary determinant of biological activity may not be the binding stability itself, but rather other contributing factors. These may include specific interactions with key residues, the three-dimensional shape of the ligand, or particular electronic and thermodynamic features that influence downstream conformational or functional changes in the protein [95,96], although further experimental validation will be necessary to confirm this proposed mechanism, it is important to acknowledge the inherent limitations of in silico simulations when attempting to describe or verify functional biological processes of this complexity.

This potential allosteric mechanism of functional inhibition represents a promising novel strategy for anti-plasmid activity, offering an alternative approach to combat antimicrobial resistance. By preventing the horizontal transfer of resistance genes, this strategy could significantly reduce the rate of dissemination of multidrug-resistant microorganisms. Furthermore, inhibiting plasmid replication may lead to the eventual loss of extrachromosomal genetic material, which is not integrated into the bacterial chromosome. Over time, this could result in a progressive re-sensitization of bacterial populations to antibiotics that had previously lost efficacy, restoring the therapeutic potential of existing antimicrobial agents.

Despite the advantages of in silico methodologies, these approaches cannot fully replicate the complexity of the biological environment during an infection. Computational simulations do not account for interactions between the drug and various substances produced by the host immune system or by the bacteria themselves. Furthermore, the activity of these compounds may vary with structural changes caused by mutations, potentially altering binding mechanisms and complex formation. For these reasons, it is essential to validate the findings presented here through in vitro and in vivo experiments to confirm the efficacy of the proposed compounds. It is also important to consider the potential collateral effects that may arise when structural alterations disrupt the various functions of this protein. Several cellular processes rely on proper DNA architecture and the coordinated assembly of multiprotein complexes. For example, site-specific recombination, the initiation and synchronization of chromosomal replication at oriC, and global transcriptional regulation all depend on the structural integrity and dynamic behavior of this protein. In addition, changes that affect nucleoid organization or DNA topology could further influence essential cellular processes. Such collateral alterations may ultimately lead to modifications in bacterial physiology, potentially altering their typical interactions and behavior within the environment [97,98]. Nevertheless, the results obtained from these simulations provide a valuable foundation for the IHF development of future drugs with potential clinical applications [99,100].

## 4. Materials and Methods

### 4.1. Database and Calculation of Descriptors

To construct the dataset for training the QSAR machine learning model, an initial set of 366 compounds was selected from a previously published study that investigated antimicrobial and anti-plasmid activity through high-throughput screening of over 12,000 known bioactive molecules. Following a detailed curation process, 65 compounds were retained. This selection excluded duplicates, compounds not indexed in major chemical databases, and those lacking retrievable structural information. The 2D chemical structures of the selected compounds were retrieved from the PubChem database. To ensure accurate geometries for descriptor calculation, energy minimization was performed using GaussView version 6 [101], applying Density Functional Theory (DFT) with the B3LYP functional and the 6–31G(d) basis set. Subsequently, molecular descriptors including both topological and electronic properties were calculated using the QuBiLs-MIDAS software https://tomocomd.com/software (accessed on 10 June 2025). These descriptors served as input features for the QSAR model. To prepare the biological activity data, the experimental values expressed as the RFU/OD600 ratio were log-transformed. This normalization ensured that higher values reflected greater biological activity, thereby enhancing data interpretability and improving the robustness of statistical analyses during model development.

### 4.2. Building and Analysis of the QSAR

Once the dataset was compiled into a .csv file containing the 65 selected compounds, their corresponding biological activity values, and the full set of calculated molecular descriptors, it was subjected to a preprocessing pipeline using a custom script. The first step involved eliminating autocorrelated variables, which helped reduce multicollinearity, minimize redundancy and noise, and improve the model’s interpretability. Subsequently, columns containing missing or null values were removed. After this cleaning process, approximately 16,000 descriptors remained for further analysis. From this refined set, only the descriptors showing the strongest correlation with the dependent variable (biological activity) were selected for model training.

A linear regression model was developed, yielding a predictive equation that estimates plasmid inhibition activity from molecular features. To ensure the robustness and reliability of the model, several validation techniques were employed, including Hold-Out, 10-Fold Cross Validation, Leave-One-Out Cross Validation, and Repeated K-Fold. For each validation method, key performance metrics were calculated: R^2^, Q^2^, Root Mean Square Error of Prediction (RMSEP), and Mean Absolute Error (MAE). The validity of the model was further confirmed using Tropsha’s criteria and Y-scrambling tests, which assess the risk of chance correlations. Finally, the applicability domain of the model was defined using Interquartile Range (IQR) and Cook’s Distance, ensuring that predictions are made only within the chemical space where the model is considered reliable [102].

### 4.3. Selection of Representative Compound and Target Identification

The 3D structures of the molecules used to train the QSAR model were subjected to an automated pharmacophoric clustering process, using structures stored in .sdf format. Through an iterative procedure, all molecules were loaded, and pharmacophores were generated using the pmapper Python library. Subsequently, various fingerprints representing key structural features of the molecules were computed using RDKit. To improve the accuracy of structural comparisons, molecular planarity alignment was performed. After this step, Tanimoto similarity scores [103,104] were applied within each cluster to identify the compound most representative of its group. The largest cluster contained 31 compounds, and its representative molecule was ICCB-3.

This compound was used to identify potential molecular targets through PharmMapper, a well-established web-based platform for reverse pharmacophore mapping. This tool identifies likely biological targets for small molecules by comparing their pharmacophoric features against a comprehensive database of experimentally validated protein-ligand interactions. PharmMapper utilizes a diverse collection of pharmacophore models derived from natural ligands, known drug targets, and repurposed therapeutic agents. By aligning the pharmacophoric features of the query compound with those in its database, the platform predicts potential protein targets with high confidence. This approach allows for the identification of plausible molecular interactions without requiring prior knowledge of the compound’s biological activity, highlighting the predictive strength and versatility of the software.

### 4.4. Docking and Molecular Dynamics Protocol

Molecular docking was performed using the E. coli Integration Host Factor (IHF) structure crystallized in complex with DNA (PDB 1IHF, 2.5 Å). This structure captures the characteristic DNA U-turn generated by IHF, in which Pro65 and neighboring residues intercalate into the minor groove and initiate sharp bending of the DNA duplex, thereby defining the native DNA-binding interface of the protein. The DNA-binding site used in this study was defined directly according to the residue–DNA contacts observed in the 1IHF crystal structure, following the binding architecture described by Rice et al. [105]. For docking, the DNA strands were removed while preserving the protein conformation adopted in the DNA-bound state. All crystallographic water molecules and non-protein or non-DNA heteroentities were removed prior to receptor preparation. Hydrogen atoms were added, and protonation states were assigned at pH 7.4. Two docking grids were created: (i) one centered on the experimentally defined DNA-binding site from 1IHF, at X: 2, Y: 14, Z: 4 with dimensions of 50 Å × 50 Å × 50 Å and (ii) a second grid positioned at a putative allosteric pocket identified using fpocket and CB-Dock2, with centered at X: −2, Y: −9, Z: 34 with the same dimensions for the cubid grid (50 Å × 50 Å × 50 Å). Both grids were used for exhaustive sampling of ligand conformations.

All docking calculations were carried out in flexible-ligand mode, i.e., with full torsional freedom for each compound throughout the search. This choice is particularly relevant for allosteric evaluation, because ligand adaptability often underpins binding at cryptic or partially formed pockets. All docking simulations were performed using AutoDockGPU, which employs the ADADELTA optimization algorithm and an enhanced local search method, enabling extensive conformational sampling through GPU acceleration. For each compound, 1000 conformations were generated, with scoring parameters set to 2,500,000 evaluations and a maximum of 42,000 generations. Data analysis was conducted using a custom script that extracted the best binding scores, calculated the mean score across all conformations, and determined the standard deviation and RMSD for each cluster of generated complexes (Supplementary Material S1).

Molecular dynamics (MD) simulations were performed using YASARA Structure over a period of 200 nanoseconds, employing the AMBER14 force field. A cubic simulation box was defined with a 5 Å margin around all atoms of the complex, ensuring complete coverage of the system, and periodic boundary conditions were applied to maintain continuity throughout the simulation. Long-range electrostatic interactions were calculated using the Particle-Mesh Ewald method. Physiological conditions were simulated by setting pH to 7.4, ionic strength to 0.9% NaCl, temperature at 298 K, and adjusting pressure and water density dynamically to maintain standard values. The water density was kept at 0.997 g/mL using the TIP3P solvation model. Post-simulation analysis was conducted using two YASARA macros; the first calculated structural and dynamic parameters, including RMSD, RMSF, radius of gyration, solvent-accessible surface area (SASA), and dynamic cross-correlation matrices (DCCM). The second estimated binding energy between receptor and ligand, using an MM/PBSA-like approach, incorporates an approximation of the entropic cost associated with solvent exposure. This calculation considers that water molecules in contact with the protein surface reorganize themselves and lose conformational freedom, reducing entropy and increasing energy cost. A coefficient of 0.65 was applied to account for Van der Waals and long-range interactions between the protein surface and water molecules. All results were compiled and tabulated for subsequent statistical analysis. The 2D interaction diagrams from docking and molecular dynamics simulations were generated using Discovery Studio Visualizer for subsequent analysis https://discover.3ds.com (accessed on 20 September 2025).

## 5. Conclusions

The computational workflow developed in this study, integrating QSAR-based machine learning (R^2^ = 0.90), molecular docking, and 200 ns molecular dynamics simulations, identified a promising allosteric mechanism for inhibiting plasmid transfer in bacteria. This mechanism targets the Integration Host Factor (IHF), a protein essential for initiating plasmid replication and mobilization. Pharmacophore-based screening selected ICCB-3 as a representative compound, and reverse pharmacophore mapping using PharmMapper supported IHF as its potential molecular target. Docking analysis showed that compounds binding to the allosteric site generally exhibited more favorable binding energies than those acting through direct inhibition. For example, ICCB-26 reached −12.15 kcal/mol, while ICCB-53 scored −11.62 kcal/mol; these values correspond specifically to interactions within the proposed allosteric pocket. However, the variation in APII values among compounds with similar binding energies suggests that biological activity is not determined solely by affinity. This indicates that additional factors-such as structural rearrangements induced upon ligand binding-likely contribute to the mechanism of action. Molecular dynamics simulations confirmed the stability and favorable energetics of these complexes over 200 ns, supported by RMSD, RMSF, radius of gyration, SASA, and binding energy analyses. Dynamic cross-correlation analysis revealed that ligand binding at the allosteric site induces conformational changes that weaken IHF–DNA interactions, particularly involving residues Phe52 and Leu26, which in turn affect the positioning of Pro65 and other residues critical for DNA binding. These structural alterations may hinder the assembly required for plasmid replication, reducing plasmid copy numbers, and limiting both horizontal and vertical gene transfer. Such inhibition represents a promising strategy to curb the dissemination of antibiotic resistance and restore bacterial susceptibility to previously ineffective antibiotics. While these computational findings are encouraging, experimental validation through in vitro and in vivo studies is essential to confirm the proposed mechanism and advance these compounds toward preclinical and clinical evaluation.

## Figures and Tables

**Table 1:** Anti-Plasmid Inhibition Index (APII) values and corresponding Compound Identifiers (CIDs) for the molecules in the QSAR training dataset.

ICCB-ID	Trivial-NAME	CID	APII	ICCB-ID	Trivial-NAME	CID	APII
ICCB-1	Bromfenac	60726	0.347	ICCB-34	Lonidamine	39562	0.569
ICCB-2	NPPB	4549	0.553	ICCB-35	MG-132	462382	0.409
ICCB-3	8-Hydroxydaidzein	5466139	0.367	ICCB-36	MK-2461	44137946	0.733
ICCB-4	AZD8055	25262965	0.347	ICCB-37	Alisertib	24771867	0.382
ICCB-5	Amorolfine	54260	0.648	ICCB-38	Laselipag	9931891	0.328
ICCB-6	Aripiprazole	60795	0.403	ICCB-39	FG 7142	4375	0.561
ICCB-7	Azacitidine	9444	0.530	ICCB-40	BRN 2143451	201986	0.530
ICCB-8	MS-27330	6914573	0.523	ICCB-41	SCHEMBL9910611	6178305	0.545
ICCB-9	Baicalein	5281605	0.469	ICCB-42	Oxaprozin	4614	0.367
ICCB-10	CFTRinh 172	1554210	0.362	ICCB-43	Proflavine	7099	0.444
ICCB-11	Cinchophen	8593	0.352	ICCB-44	Pentamidine	4735	0.658
ICCB-12	Carprofen	2581	0.553	ICCB-45	Hezamidine	65130	0.642
ICCB-13	CH55	6184667	0.530	ICCB-46	Piceatannol	667639	0.638
ICCB-14	Chrysin	5281607	0.450	ICCB-47	Pipemidic acid	4831	0.611
ICCB-15	DCPIB	10071166	0.415	ICCB-48	Pro-Banthine	9279	0.432
ICCB-16	CHEMBL1618718	5378825	0.415	ICCB-49	Quinalizarin	5004	0.319
ICCB-17	Dequalinium	2993	0.409	ICCB-50	AC1LCVGT	656717	0.367
ICCB-18	Diflunisal	3059	0.495	ICCB-51	Rifabutina	135415564	0.420
ICCB-19	Dobutamine	36811	0.561	ICCB-52	SC58125	115239	0.415
ICCB-20	Efloxate	8395	0.382	ICCB-53	SH-4-54	72188643	0.310
ICCB-21	FG 7142	4375	0.495	ICCB-54	Lintitript	122077	0.393
ICCB-22	Febuxostat	134018	0.481	ICCB-55	STX-0119	4253236	0.502
ICCB-23	Genistein	5280961	0.398	ICCB-56	Sertaconazole	200103	0.456
ICCB-24	Glyburide	3488	0.577	ICCB-57	Shikonin	479503	0.409
ICCB-25	Glimepiride	3476	0.530	ICCB-58	DTXSID60587903	16760513	0.438
ICCB-26	Gliquidone	91610	0.668	ICCB-59	TPCA-1	9903786	0.469
ICCB-27	Icatibant	6918173	0.382	ICCB-60	Terbinafine	1549008	0.387
ICCB-28	Idoxuridine	5905	0.481	ICCB-61	Tipifarnib	159324	0.495
ICCB-29	Imperatorin	10212	0.377	ICCB-62	Toceranib	5329106	0.456
ICCB-30	Isoliquiritigenin	638278	0.577	ICCB-63	402-71-1	439647	0.409
ICCB-31	Kasugamycin	65174	0.620	ICCB-64	UK-383367	9818682	0.403
ICCB-32	LY-255283	122023	0.561	ICCB-65	Vidofludimus	9820008	0.509
ICCB-33	Lincomycin	64710	0.415				

**Table 2:** Molecular descriptors and their corresponding regression coefficients contribute to the construction of the 3D-QSAR model.

Code	Descriptor	Coefficient (β)	R^2^ Contributions
	Intercept	0.6445	
X1	I50_B_AB_nCi_2_M3_SS1_T_KA_h−s_MID	−1.5200	22.02%
X2	AC[4]_K_F_AB_Ci(−1.25;1.25)_2_M10_MP0_T_LGP[2]_psa_MID	−0.0038	19.35%
X3	IB_S_B_AB_nCi_2_M3_NS0_M_LGL[2–3]_a_p_MID	−0.0203	26.10%
X4	AC[1]_S_B_AB_nCi_2_M3_NS0_T_LGP[2]_e_p_MID	−0.0363	9.32%
X5	AC[3]_K_F_AB_Ci(0.25;−0.25)_2_M15_MP1_X_KA_c_MID	0.0038	9.60%
X6	MN_TrC_AB_nCi_3_M20(M16)_SS2_T_LG3P[1]_LGP[1]_h_MID	6.0267	6.77%
X7	IB_S_F_AB_Ci(0.25;−0.25)_2_M10_SS0_X_KA_c_MID	0.0046	6.84%

**Table 3:** Statistical parameters of the QSAR model developed for steroidal compounds exhibiting inhibitory activity against plasmid transfer.

Metric	OLS-Hold-Out	LOOCV
R (Correlation Coefficient)	0.9673	0.9076
R^2^ (Determination Coefficient)	0.9034	0.8232
R^2^_ext_	0.9034	NaN
Q^2^ (Predictive Squared Correlation)	NaN	0.8232
QMRF (Mean Squared Prediction Error)	0.0014	0.0017
RMSEP (Square Root of MSE)	0.0378	0.0413
MAEext	0.0283	0.0337
(R^2^ − R_0_^2^)/R^2^ < 0.1	0.0354	0.0000
(R^2^ − R_0_′^2^)/R^2^ < 0.1	0.0277	0.0313
|R_0_^2^ − R_0_′^2^| < 0.1	0.0070	0.0258
0.85 < K < 1.15	1.0466	0.9992
0.85 < k’ < 1.15	0.9517	0.9935

**Table 4:** Docking binding energies (ΔG, in kcal/mol) calculated for both direct and allosteric inhibition modes of the QSAR training set compounds when docked against the Integration Host Factor (IHF) protein (PDB ID: 1IHF).

Direct Inhibition									
ICCB_ID	Mean	BestEnergy	DesvSt	RMSD	ICCB_ID	Mean	BestEnergy	DesvSt	RMSD
ICCB-3	−6.06	−6.22	0.06	1.96	ICCB-38	−6.42	−7.1	0.27	1.81
ICCB-4	−7.83	−8.01	0.08	1.33	ICCB-43	−5.27	−5.28	0.00	0.05
ICCB-7	−6.28	−6.64	0.17	0.43	ICCB-44	−6.38	−6.92	0.27	0
ICCB-8	−7.36	−7.89	0.22	1.76	ICCB-45	−7.80	−8.23	0.2	0.46
ICCB-9	−6.38	−6.47	0.07	1.76	ICCB-47	−7.46	−7.81	0.18	1.85
ICCB-15	−6.89	−7.29	0.27	0.88	ICCB-49	−7.01	−7.07	−6.97	0.45
ICCB-23	−6.47	−6.69	0.15	0.3	ICCB-51	−9.31	−9.57	0.14	0.46
ICCB-24	−6.47	−6.69	0.15	1.63	ICCB-53	−8.48	−9.49	0.32	1
ICCB-25	−8.07	−8.69	0.2	1.75	ICCB-54	−7.34	−7.88	0.26	1.18
ICCB-26	−8.68	−9.41	0.31	0	ICCB-55	−8.26	−8.42	0.07	0.28
ICCB-27	−7.89	−8.47	0.26	0.98	ICCB-56	−6.81	−7.96	0.32	1.37
ICCB-31	−7.63	−7.96	0.17	1.21	ICCB-58	−7.63	−7.98	0.16	1.64
ICCB-32	−6.35	−7.07	0.34	0.83	ICCB-61	−7.02	−7.41	0.16	1.48
ICCB-33	−7.67	−7.94	0.16	0.87	ICCB-62	−7.24	−7.54	0.12	1.71
ICCB-36	−8.55	−9.46	0.34	1.93	ICCB-65	−7.26	−7.79	0.18	1.15
ICCB-37	−8.06	−8.41	0.16	−7.67					
**Allosteric Inhibition**									
**ICCB_ID**	**Mean**	**BestEnergy**	**DesvSt**	**RMSD**	**ICCB_ID**	**Mean**	**BestEnergy**	**DesvSt**	**RMSD**
ICCB-3	−7.47	−7.49	0.01	0.13	ICCB-38	−7.98	−8.23	0.14	0.88
ICCB-4	−10.2	−10.26	0.04	0.4	ICCB-43	−7.06	−7.12	0.04	0.23
ICCB-7	−7.3	−7.39	0.02	0.84	ICCB-44	−8.17	−8.48	0.14	1.01
ICCB-8	−8.95	−9.25	0.14	1.8	ICCB-45	−10.39	−10.74	0.17	1.26
ICCB-9	−7.35	−7.41	0.06	0.15	ICCB-47	−8.15	−8.28	0.08	0.31
ICCB-15	−7.74	−8.21	0.16	0.86	ICCB-49	−7.26	−7.36	0.05	0.49
ICCB-23	−7.1	−7.34	0.06	0.9	ICCB-51	−10.26	−10.62	0.16	0.66
ICCB-24	−10.59	−11.27	0.23	1.04	ICCB-53	−11.34	−11.62	0.18	1.08
ICCB-25	−11	−11.36	0.14	0.98	ICCB-54	−8.83	−9.12	0.12	0.63
ICCB−26	−11.58	−12.15	0.22	1.12	ICCB-55	−10.25	−10.29	0.02	0.48
ICCB-27	−7.88	−8.96	0.7	1.14	ICCB-56	−8.86	−8.73	0.13	1.38
ICCB-31	−8.2	−8.91	0.26	0.76	ICCB-58	−9.85	−10.07	0.11	0.60
ICCB-32	−7.68	−8.84	0.48	1.58	ICCB-61	−9.22	−9.64	0.06	1.70
ICCB-33	−8.59	−8.36	0.16	1.16	ICCB-62	−9.64	−10.04	0.15	1.60
ICCB-36	−10.38	−10.61	0.13	1.64	ICCB-65	−8.86	−9.18	0.16	1.75
ICCB-37	−9.73	−10.24	0.25	1.8					

## Data Availability

The original contributions presented in this study are included in the article/Supplementary Material. Further inquiries can be directed to the corresponding author.

## Supplementary Materials

The following supporting information can be downloaded at: https://www.mdpi.com/article/10.3390/ijms27062526/s1.
