# Supplementary material for: Computational Identification of Potential Novel Allosteric IHF Inhibitors Using QSAR Modeling to Inhibit Plasmid-Mediated Antibiotic Resistance
Source: Int J Mol Sci. 2026 Mar 10;27(6):2526. doi: 10.3390/ijms27062526 (PMC13026255; doi:10.3390/ijms27062526)
Supplement: Supplementary file 1 [file ijms-27-02526-s001.zip › ijms-4143930-supplementary.pdf]

Computational Identification of Potential Novel Allosteric IHF Inhibitors Using QSAR Modeling to Inhibit Plasmid-Mediated Antibiotic Resistance

Oscar Saurith-Coronell [1,2] , Olimpo Sierra-Hernandez [1,2] , Juan David Rodríguez-Macías [3,*] , José R. Mora [4] , Noel Perez-Perez [5] , Jackson J. Alcázar [6] , Ricardo Olimpio de Moura [7] , Igor José dos Santos Nascimento [7] , Edgar A. Márquez Brazón [2,*] and Yovani Marrero-Ponce [8,9]

Supplementary Material

Table S1: Descriptors incorporated into the predictive QSAR model, along with their corresponding values for each molecule, are provided.

molecules	I50_B_AB_nCi_2_M3_SS1_T_KA_h-s_MID	AC[1]_S_B_AB_nCi_2_M3_NS0_T_LGP[2]_e-p_MID	IB_S_F_AB_Ci(0.25;-0.25)_2_M10_SS0_X_KA_c_MID	AC[3]_K_F_AB_Ci(0.25;-0.25)_2_M15_MP1_X_KA_c_MID	MN_TrC_AB_nCi_3_M20(M16)_SS2_T_LG3P[1]_LGP[1]_h_MID	AC[4]_K_F_AB_Ci(-1.25;1.25)_2_M10_MP0_T_LGP[2]_psa_MID	IB_S_B_AB_nCi_2_M3_NS0_M_LGL[2-3]_a-p_MID
ICCB36	0.07	0.05	3.99	13.60	0.00	0.57	-4.36
ICCB26	0.05	0.37	-0.14	12.43	0.00	6.60	-4.29
ICCB44	0.05	-0.48	1.99	11.65	0.00	-3.99	0.00
ICCB45	0.05	-0.48	1.99	11.65	0.00	-3.99	0.00
ICCB5	0.07	-0.09	0.88	6.01	0.01	7.35	-2.92
ICCB46	0.05	0.07	2.49	3.41	0.00	0.00	0.00
ICCB31	0.07	0.54	1.99	-0.04	0.00	1.68	-3.80

ICCB47	0.11	0.16	2.53	-0.82	0.00	3.90	-3.53
ICCB24	0.08	0.29	1.01	12.10	0.00	3.34	0.00
ICCB30	0.09	0.42	1.91	4.45	0.00	6.82	0.00
ICCB34	0.10	0.53	0.68	5.65	0.00	4.53	0.00
ICCB19	0.05	-0.28	2.77	3.49	0.00	0.65	3.53
ICCB32	0.09	0.27	1.06	16.52	0.00	15.28	-3.68
ICCB39	0.11	0.06	-1.39	0.93	0.00	-3.65	0.00
ICCB2	0.08	0.92	-2.13	8.92	0.00	3.06	0.00
ICCB12	0.10	0.22	-0.05	18.94	0.00	11.60	0.00
ICCB41	0.11	0.28	-2.50	0.55	0.00	1.65	0.00
ICCB7	0.11	0.56	1.12	0.21	0.01	3.20	0.00
ICCB13	0.10	0.92	1.81	0.93	0.00	0.00	-2.29
ICCB25	0.06	0.25	0.93	15.88	0.00	2.93	2.39

ICCB40	0.08	0.92	-2.13	8.92	0.00	3.06	0.00
ICCB8	0.08	0.39	1.03	10.53	0.00	10.84	0.00
ICCB65	0.08	-0.20	-0.40	-0.64	0.00	0.00	0.00
ICCB55	0.08	0.47	-2.55	1.76	0.00	10.13	0.00
ICCB18	0.10	0.41	0.36	11.15	0.00	11.37	0.00
ICCB21	0.11	0.06	-1.39	0.93	0.00	-3.65	0.00
ICCB61	0.10	0.47	-0.69	1.65	0.00	24.26	-3.71
ICCB22	0.08	-0.06	0.94	-0.05	0.00	6.21	1.72
ICCB28	0.07	0.34	0.53	0.39	0.00	3.01	0.00
ICCB9	0.09	0.46	1.49	-1.14	0.00	12.27	0.00
ICCB59	0.11	0.34	2.64	3.55	0.00	4.17	0.00
ICCB56	0.08	0.47	1.77	-0.44	0.00	12.53	0.00
ICCB62	0.10	0.16	-0.83	1.90	0.00	27.97	-3.81

ICCB14	0.12	0.53	1.05	-0.93	0.00	11.37	0.00
ICCB43	0.11	0.06	-0.49	-1.03	0.00	13.47	0.00
ICCB58	0.07	0.80	-2.86	7.09	0.00	7.68	0.00
ICCB48	0.09	0.83	3.31	1.11	0.00	22.15	-3.67
ICCB51	0.12	0.93	3.72	8.28	0.00	3.59	3.29
ICCB15	0.12	0.31	0.99	6.95	0.00	21.34	0.00
ICCB16	0.08	1.03	0.30	0.10	0.00	5.16	0.00
ICCB33	0.10	0.45	1.14	1.99	0.00	11.25	3.75
ICCB52	0.09	1.09	3.30	4.95	0.00	1.13	3.80
ICCB17	0.10	0.62	-0.01	11.72	0.00	2.33	3.00
ICCB35	0.06	0.58	-3.82	2.67	0.00	4.60	4.22
ICCB57	0.10	0.47	0.70	-0.66	0.00	13.61	0.00
ICCB63	0.11	0.67	2.58	4.36	0.00	1.89	3.61

ICCB6	0.08	0.96	-3.03	1.21	0.00	17.28	0.00
ICCB64	0.11	0.81	-1.16	1.19	0.00	15.96	0.00
ICCB23	0.10	0.24	1.27	-0.51	0.00	15.94	0.00
ICCB54	0.11	0.20	2.22	1.36	0.00	17.40	0.00
ICCB60	0.09	0.75	-0.42	3.11	0.00	27.13	2.89
ICCB20	0.12	0.51	-3.21	-1.29	0.00	9.25	3.67
ICCB27	0.10	1.05	-5.65	3.60	0.00	14.68	0.00
ICCB37	0.08	0.83	-3.70	6.34	0.00	12.81	3.61
ICCB29	0.10	0.13	-2.59	-1.00	0.00	12.26	0.00
ICCB3	0.12	0.21	1.52	-0.69	0.00	15.49	0.00
ICCB42	0.13	0.87	-0.59	3.10	0.00	5.94	0.00
ICCB50	0.10	0.03	-0.56	12.49	0.00	13.09	3.53
ICCB10	0.09	1.27	-0.60	1.13	0.00	19.67	0.00

ICCB11	0.15	0.33	-1.27	0.26	0.00	6.66	0.00
ICCB1	0.13	-0.06	-2.55	0.97	0.00	29.12	0.00
ICCB4	0.10	0.47	-0.97	0.18	0.00	15.67	4.21
ICCB38	0.13	0.83	-3.72	6.61	0.00	4.68	3.50
ICCB49	0.13	0.54	0.47	-0.74	0.00	5.96	0.00
ICCB53	0.07	0.59	-0.75	2.37	0.00	39.53	2.83

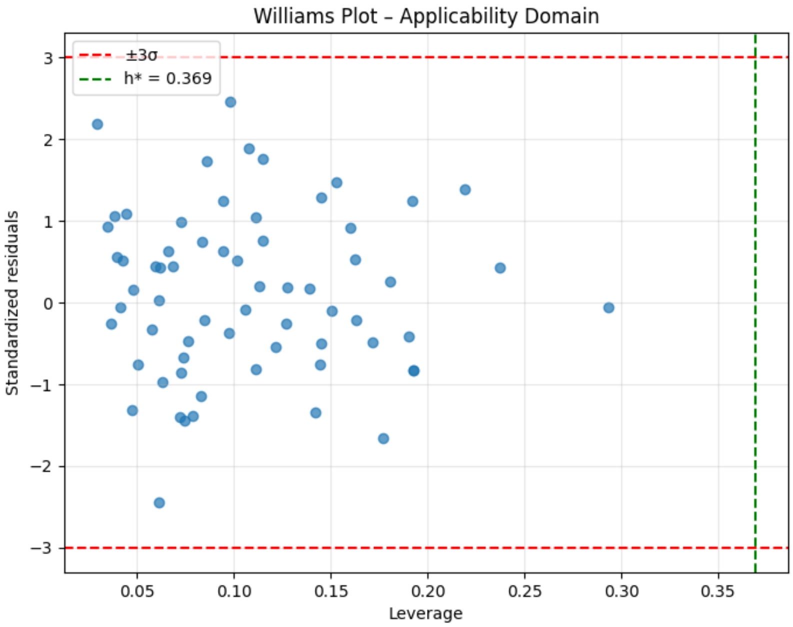

Figure S1. Williams Plot showing standardized residuals versus leverage values. Most compounds lie within ±3 residual limits and below the leverage threshold, indicating a reliable applicability domain.

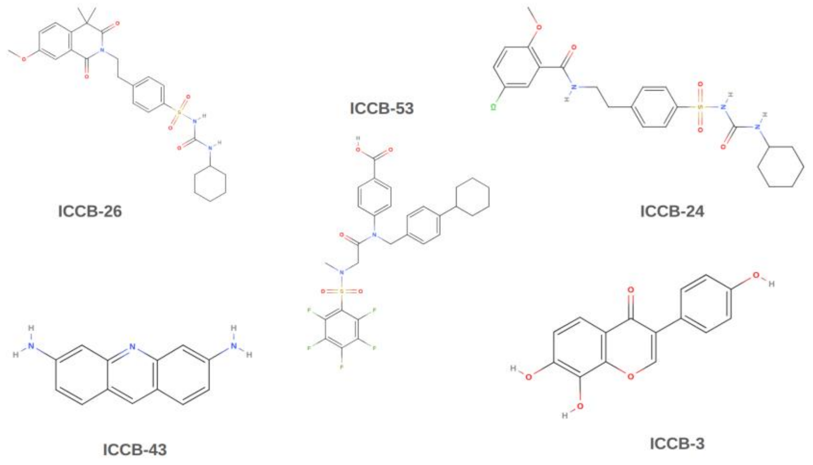

Figure S2. Chemical structures of the compounds selected as representatives for docking and molecular dynamics simulations are shown.

Supplementary material, S1. Script for the Analysis of Docking Simulation Results

#!/bin/bash

et -e

echo "===" echo " ANÁLISIS COMPLETO DE RESULTADOS " echo "==="

DIRECTORIO_BASE="/home/ChemFusion"
DIRECTORIO_RESULTADOS="$DIRECTORIO_BASE/Resultados"
DIRECTORIO_DOCKING="$DIRECTORIO_RESULTADOS/ResultadosDocking"
DIRECTORIO_DINAMICA="$DIRECTORIO_RESULTADOS/Dinamica"
DIRECTORIO_ARCHIVOS_DOCKING="$DIRECTORIO_RESULTADOS/ArchivosDocking"

Rutas de aplicaciones - CONFIGURACIÓN MGLTOOLS MEJORADA

MGLTOOLS_PATH="/home/ChemFusion/funciones/Aplicaciones/MGLTools-1.5.6"
MGLTOOLS_PYTHON="$MGLTOOLS_PATH/bin/python"
PDBQT_TO_PDB_SCRIPT="$MGLTOOLS_PATH/MGLToolsPckgs/AutoDockTools/Utilities24/pdbqt_to_pdb.py"
LOWEST_ENERGY_SCRIPT="$MGLTOOLS_PATH/MGLToolsPckgs/AutoDockTools/Utilities24/write_lowest_energy_ligand.py"

setup_mgltools_environment() { export PYTHONPATH="$MGLTOOLS_PATH/MGLToolsPckgs:$PYTHONPATH" export MGL_ROOT="$MGLTOOLS_PATH" export PATH="$MGLTOOLS_PATH/bin:$PATH" export LD_LIBRARY_PATH="$MGLTOOLS_PATH/lib:$LD_LIBRARY_PATH" }

run_mgltools_command() { local cmd="$1" shift

```
# Guardar entorno actual
local old_pythonpath="$PYTHONPATH"

local old_mgl_root="$MGL_ROOT"
local old_path="$PATH"
local old_ld_library_path="$LD_LIBRARY_PATH"

# Configurar entorno MGLTools
setup_mgltools_environment

# Ejecutar comando
"$MGLTOOLS_PYTHON" "$cmd" "$@"
local exit_code=$?

# Restaurar entorno (opcional, pero buena práctica)
export PYTHONPATH="$old_pythonpath"
export MGL_ROOT="$old_mgl_root"
export PATH="$old_path"
export LD_LIBRARY_PATH="$old_ld_library_path"

return $exit_code
```

}

extraer_nombre_molecula_mejorado() { local archivo="$1" local nombre_base=$(basename "$archivo")

```
# Método 1: Buscar patrón después de proteína preparada
if [[ "$nombre_base" =~ _prepared_(.+)\.xml$ ]]; then
   echo "${BASH_REMATCH[1]}"
   return 0
fi

# Método 2: Buscar último componente antes de .xml
if [[ "$nombre_base" =~ _([^_]+)\.xml$ ]]; then
   echo "${BASH_REMATCH[1]}"
   return 0
fi

# Método 3: Remover extensión y tomar último componente
```

```
local sin_extension="${nombre_base%.xml}"
if [[ "$sin_extension" =~ _([^_]+)$ ]]; then
   echo "${BASH_REMATCH[1]}"
   return 0
fi

# Fallback: usar nombre completo sin extensión
echo "$sin_extension"


}

extraer_binding_energies() { local archivo_xml="$1"

# Método mejorado para extraer energías de binding
# Primero intentamos con el método tradicional
local binding_energies=$(grep -o '<free_NRG_binding>.*</free_NRG_binding>' "$archivo_xml" | \
                  sed 's/<free_NRG_binding>//g' | \
                  sed 's/<\/free_NRG_binding>//g' | \
                  tr -d ' \t\n\r' | \
                  grep -E '^-?[0-9]+\.?[0-9]*$')

# Si no se encontraron energías, intentamos un método más flexible
if [ -z "$binding_energies" ]; then
   binding_energies=$(grep -i "free_NRG_binding" "$archivo_xml" | \
                 sed 's/.*<free_NRG_binding>//g' | \
                 sed 's/<\/free_NRG_binding>.*//g' | \
                 tr -d ' \t\n\r' | \
                 grep -E '^-?[0-9]+\.?[0-9]*$')
fi

# Si aún no se encuentran, buscamos cualquier valor numérico en el contexto de binding
if [ -z "$binding_energies" ]; then
   binding_energies=$(grep -i "binding" "$archivo_xml" | \
                 grep -Eo '[-+]?[0-9]*\.?[0-9]+' | \
                 head -20)  # Limitar para no capturar demasiados valores
fi

echo "$binding_energies"


}

function generar_reporte_completo_binding_energy() { local directorio_dat="$1" local
directorio_analisis="$directorio_dat/AnalisisDocking"

echo "Generando reporte completo de binding energy para todas las moléculas..."
local archivo_reporte_binding="$directorio_analisis/Reporte_BindingEnergy_Completo.txt"
```

```
{
    echo "=============================================="
    echo "REPORTE COMPLETO DE BINDING ENERGY - $(date)"
    echo "=============================================="
    echo ""

    # Buscar archivos XML y crear mapa único
    declare -A archivos_procesados
    declare -A xml_por_molecula

    # Mapear archivos XML únicos por molécula
    while IFS= read -r -d '' archivo_xml; do
        local nombre_molecula=$(extraer_nombre_molecula_mejorado "$archivo_xml")

        # Limpiar caracteres problemáticos
        nombre_molecula=$(echo "$nombre_molecula" | tr -d ' \t\n\r' | sed 's/[^a-zA-Z0-9_-]/_/g')

        # Solo procesar si el nombre no está vacío y no es solo símbolos
        if [[ -n "$nombre_molecula" && "$nombre_molecula" != "_" && "$nombre_molecula" != "#" ]];
then
            # Si ya tenemos un XML para esta molécula, usar el más reciente
            if [[ -z "${xml_por_molecula[$nombre_molecula]}" ]] || [[ "$archivo_xml" -nt
"${xml_por_molecula[$nombre_molecula]}" ]]; then
                xml_por_molecula["$nombre_molecula"]="$archivo_xml"
            fi
        fi
    done < <(find "$directorio_dat" -type f -name "*.xml" -print0)

    local total_moleculas_unicas=${#xml_por_molecula[@]}

    echo "Moléculas únicas encontradas: $total_moleculas_unicas"
    echo ""

    if [ $total_moleculas_unicas -eq 0 ]; then
        echo "No se encontraron archivos XML válidos para analizar."
        return 1
    fi

    # Variables para estadísticas globales
    local todos_binding_energies=""
    local total_runs_global=0
    local moleculas_procesadas=0

    echo "ANÁLISIS POR MOLÉCULA:"
    echo "====================="
    echo ""
```

```
# Arrays para ranking
declare -a promedios_exactos
declare -a nombres_moleculas_ranking

# Procesar cada molécula única
for nombre_molecula in "${!xml_por_molecula[@]}"; do
   local archivo_xml="${xml_por_molecula[$nombre_molecula]}"
   local nombre_archivo=$(basename "$archivo_xml")

   echo "MOLÉCULA: $nombre_molecula"
   echo "    Archivo XML: $nombre_archivo"

   # Contar runs en este XML
   local runs_molecula=$(grep -c '<run id=' "$archivo_xml" 2>/dev/null || echo "0")
   total_runs_global=$((total_runs_global + runs_molecula))

   echo "    Corridas encontradas: $runs_molecula"

   if [ $runs_molecula -gt 0 ]; then
      # Extraer binding energies de este XML usando la función mejorada
      local binding_energies_molecula=$(extraer_binding_energies "$archivo_xml")

      if [ -n "$binding_energies_molecula" ]; then
         # Agregar a la colección global
         if [ -z "$todos_binding_energies" ]; then
            todos_binding_energies="$binding_energies_molecula"
         else
            todos_binding_energies="$todos_binding_energies


$binding_energies_molecula" fi

         # Calcular estadísticas para esta molécula
         local stats_molecula=$(echo "$binding_energies_molecula" | LC_NUMERIC=C awk '
            BEGIN { sum = 0; count = 0; most_positive = ""; most_negative = "" }
            /^-?[0-9]+\.?[0-9]*$/ {
               values[count] = $1
               sum += $1
               count++

               if (most_positive == "") {
                  most_positive = $1
                  most_negative = $1
               } else {
                  if ($1 > most_positive) most_positive = $1
                  if ($1 < most_negative) most_negative = $1
               }
            }
```

```
          END {
            if (count > 0) {
              avg = sum/count

              sum_sq_diff = 0
              for (i = 0; i < count; i++) {
                diff = values[i] - avg
                sum_sq_diff += diff * diff
              }
              std_dev = sqrt(sum_sq_diff / count)

              printf "    Promedio: %.2f kcal/mol\n", avg
              printf "    Desviación estándar: %.2f kcal/mol\n", std_dev
              printf "    Menos favorable: %.2f kcal/mol\n", most_positive
              printf "    Más favorable: %.2f kcal/mol\n", most_negative
              printf "PROMEDIO_EXACTO:%.6f\n", avg
            } else {
              printf "    No se encontraron valores numéricos válidos\n"
              printf "PROMEDIO_EXACTO:N/A\n"
            }
          }'
        )

        # Extraer promedio exacto para ranking
        local promedio_exacto=$(echo "$stats_molecula" | grep "PROMEDIO_EXACTO:" | cut -d':'
-f2)

        # Mostrar estadísticas
        echo "$stats_molecula" | grep -v "PROMEDIO_EXACTO:"

        # Almacenar para ranking solo si es válido
        if [[ "$promedio_exacto" != "N/A" && -n "$promedio_exacto" ]]; then
          promedios_exactos+=("$promedio_exacto")
          nombres_moleculas_ranking+=("$nombre_molecula")
        fi

        moleculas_procesadas=$((moleculas_procesadas + 1))
      else
        echo "    No se encontraron valores de binding energy válidos"
        echo "    Contenido del archivo (primeras líneas):"
        head -5 "$archivo_xml" | sed 's/^/        /'
      fi
    else
      echo "    No se encontraron corridas en el archivo XML"
    fi

    echo ""
  done
```

```
# Continuar con estadísticas globales
echo "=============================================="
echo "ESTADÍSTICAS GLOBALES (TODAS LAS MOLÉCULAS)"
echo "=============================================="
echo "Moléculas procesadas exitosamente: $moleculas_procesadas"
echo "Total de corridas analizadas: $total_runs_global"
echo ""

if [ -n "$todos_binding_energies" ] && [ $moleculas_procesadas -gt 0 ]; then
  # Calcular estadísticas globales
  local stats_globales=$(echo "$todos_binding_energies" | LC_NUMERIC=C awk '
    BEGIN { sum = 0; count = 0; most_positive = ""; most_negative = "" }
    {
      values[count] = $1
      sum += $1
      count++

      if (most_positive == "") {
        most_positive = $1
        most_negative = $1
      } else {
        if ($1 > most_positive) most_positive = $1
        if ($1 < most_negative) most_negative = $1
      }
    }
    END {
      if (count > 0) {
        avg = sum/count

        sum_sq_diff = 0
        for (i = 0; i < count; i++) {
          diff = values[i] - avg
          sum_sq_diff += diff * diff
        }
        std_dev = sqrt(sum_sq_diff / count)

        printf "Promedio global: %.2f kcal/mol\n", avg
        printf "Desviación estándar global: %.2f kcal/mol\n", std_dev
        printf "Binding energy menos favorable: %.2f kcal/mol\n", most_positive
        printf "Binding energy más favorable: %.2f kcal/mol\n", most_negative
        printf "Rango: %.2f kcal/mol\n", (most_positive - most_negative)
      }
    }'
  )
  echo "$stats_globales"
fi
```

```
    # Ranking solo con moléculas válidas
    if [ ${#promedios_exactos[@]} -gt 0 ]; then
        echo ""
        echo "RANKING DE MOLÉCULAS (POR PROMEDIO DE BINDING ENERGY)"
        echo "=================================================="

        local temp_ranking=$(mktemp)

        for ((i=0; i<${#promedios_exactos[@]}; i++)); do
            echo "${promedios_exactos[i]} ${nombres_moleculas_ranking[i]}" >> "$temp_ranking"
        done

        local posicion=1
        sort -n "$temp_ranking" | while read promedio_exacto molecula; do
            local medalla=""
            case $posicion in
                1) medalla="1°" ;;
                2) medalla="2°" ;;
                3) medalla="3°" ;;
                *) medalla="  " ;;
            esac
            printf "%s %2d. %-20s %.2f kcal/mol\n" "$medalla" "$posicion" "$molecula"
"$promedio_exacto"
            posicion=$((posicion + 1))
        done

        rm -f "$temp_ranking"
    fi

    echo ""
    echo "============================================"
    echo "Los promedios individuales por molécula están incluidos en: Resultados.csv"
    echo "Este reporte completo se ha guardado en: $(basename "$archivo_reporte_binding")"
    echo "============================================"

} > "$archivo_reporte_binding"

echo "  Reporte completo de binding energy generado: $(basename "$archivo_reporte_binding")"
return 0


}
```

===

FUNCIÓN CORREGIDA: ANÁLISIS DE DATOS DE DOCKING

===

```
function analizar_datos_docking() { echo "=============================================="
echo "PASO 1: Analizando datos de docking..." echo
"=============================================="

# Buscar archivo .dat automáticamente
archivo_entrada=$(find "$DIRECTORIO_DOCKING" -type f -name "*.dat" | head -n 1)

if [ -z "$archivo_entrada" ]; then
   echo "ERROR: No se encontró ningún archivo .dat en $DIRECTORIO_DOCKING"
   return 1
fi

echo "Procesando archivo: $(basename "$archivo_entrada")"

# Obtener directorio del archivo .dat
directorio_dat=$(dirname "$archivo_entrada")

# Crear directorio para análisis
directorio_analisis="$directorio_dat/AnalisisDocking"
mkdir -p "$directorio_analisis"

archivo_resultados="$directorio_analisis/Resultados.csv"

# Ordenar datos por energía libre
echo "Ordenando resultados por energía libre..."
sort -k2,2n "$archivo_entrada" > temp_sorted.dat

# Crear mapa de moléculas únicas a XMLs
declare -A molecula_a_xml

echo "Mapeando archivos XML a nombres de moléculas..."
while IFS= read -r -d '' archivo_xml; do
   local nombre_molecula=$(extraer_nombre_molecula_mejorado "$archivo_xml")
  nombre_molecula=$(echo "$nombre_molecula" | tr -d ' \t\n\r' | sed 's/[^a-zA-Z0-9_-]/_/g')

   if [[ -n "$nombre_molecula" && "$nombre_molecula" != "_" && "$nombre_molecula" != "#" ]]; then
      if [[ -z "${molecula_a_xml[$nombre_molecula]}" ]] || [[ "$archivo_xml" -nt
"${molecula_a_xml[$nombre_molecula]}" ]]; then
         molecula_a_xml["$nombre_molecula"]="$archivo_xml"
      fi
   fi
done < <(find "$directorio_dat" -type f -name "*.xml" -print0)

echo "Moléculas únicas mapeadas: ${#molecula_a_xml[@]}"
for mol in "${!molecula_a_xml[@]}"; do
   echo "  • $mol -> $(basename "${molecula_a_xml[$mol]}")"
done
```

```
# Función mejorada para extraer binding energy
extraer_promedio_binding_energy_unico() {
  local molecula="$1"

  if [[ -n "${molecula_a_xml[$molecula]}" ]]; then
    local xml_archivo="${molecula_a_xml[$molecula]}"

    # Usar la función mejorada para extraer binding energies
    local binding_energies=$(extraer_binding_energies "$xml_archivo")

    if [[ -n "$binding_energies" ]]; then
      local promedio=$(echo "$binding_energies" | awk '
        BEGIN { sum = 0; count = 0 }
        /^-?[0-9]+\.?[0-9]*$/ {
          sum += $1
          count++
        }
        END {
          if (count > 0) {
            printf "%.2f", sum/count
          } else {
            print "N/A"
          }
        }' OFMT="%.2f"
      )

      local num_runs=$(echo "$binding_energies" | wc -l)
      echo "$promedio"
      echo "  Binding energy para $molecula: $promedio kcal/mol ($num_runs corridas)" >&2
    else
      echo "N/A"
      echo "  Valores inválidos en XML para $molecula" >&2
    fi
  else
    echo "N/A"
    echo "  XML no mapeado para $molecula" >&2
  fi
}

# Crear CSV con nombres únicos
echo -e "Molécula,EnergíaLibre(Gibbs),Pose,BindingEnergyPromedio(kcal/mol)" >
"$archivo_resultados"

# Procesar archivo .dat
declare -A moleculas_procesadas

while IFS= read -r line; do
  mol=$(echo "$line" | awk '{print $1}' | tr -d ' \t\n\r')
```

```
    energia=$(echo "$line" | awk '{print $2}' | tr ',' '.')
    pose=$(echo "$line" | awk '{print $3}')

    # Limpiar nombre de molécula
    mol_limpio=$(echo "$mol" | sed 's/[^a-zA-Z0-9_-]/_/g')

    # Skip si ya procesamos esta molécula o si es inválida
    if [[ -n "${moleculas_procesadas[$mol_limpio]}" ]] || [[ -z "$mol_limpio" ]] || [[ "$mol_limpio" ==
"_" ]]; then
        continue
    fi

    # Marcar como procesada
    moleculas_procesadas["$mol_limpio"]=1

    # Extraer binding energy
    binding_promedio=$(extraer_promedio_binding_energy_unico "$mol_limpio")
    binding_promedio=$(echo "$binding_promedio" | tr ',' '.')

    # Escribir al CSV
    echo -e "$mol_limpio,$energia,$pose,$binding_promedio" >> "$archivo_resultados"

done < temp_sorted.dat

rm temp_sorted.dat

# Generar reporte completo
generar_reporte_completo_binding_energy "$directorio_dat"

# Extraer nombres únicos para siguiente paso
echo "Extrayendo nombres únicos de moléculas..."
awk -F',' 'NR>1 {print $1}' "$archivo_resultados" | sort -u >
"$DIRECTORIO_RESULTADOS/nombres_moleculas.txt"

echo "Análisis de datos completado con nombres únicos"
echo "  - Resultados: $archivo_resultados"
echo "  - Moléculas únicas: $(wc -l < "$DIRECTORIO_RESULTADOS/nombres_moleculas.txt")"


}

function extraer_archivos_dlg() { echo "============================================" echo
"PASO 2: Extrayendo archivos DLG..." echo "============================================"

local archivo_moleculas="$DIRECTORIO_RESULTADOS/nombres_moleculas.txt"
local directorio_dlgs="$DIRECTORIO_RESULTADOS/Dlgs"

mkdir -p "$directorio_dlgs"
```

```
if [[ ! -f "$archivo_moleculas" ]]; then
   echo "ERROR: Archivo de nombres no encontrado: $archivo_moleculas"
   return 1
fi

echo "Buscando archivos DLG para moléculas únicas..."
local archivos_copiados=0

while IFS= read -r nombre_molecula; do
   if [[ -n "$nombre_molecula" && "$nombre_molecula" != "_" ]]; then
      echo "Buscando DLG para: $nombre_molecula"

      # Patrones de búsqueda más exhaustivos
      local archivos_encontrados=""

      # Patrón 1: Nombre exacto
      archivos_encontrados=$(find "$DIRECTORIO_DOCKING" -type f -path "*/dlg/*" -name
"*${nombre_molecula}*.dlg" 2>/dev/null | head -1)

      # Patrón 2: Sin espacios
      if [[ -z "$archivos_encontrados" ]]; then
         local nombre_sin_espacios=$(echo "$nombre_molecula" | tr -d ' ')
         archivos_encontrados=$(find "$DIRECTORIO_DOCKING" -type f -path "*/dlg/*" -name
"*${nombre_sin_espacios}*.dlg" 2>/dev/null | head -1)
      fi

      # Patrón 3: Con guiones bajos
      if [[ -z "$archivos_encontrados" ]]; then
         local nombre_underscore=$(echo "$nombre_molecula" | tr ' ' '_')
         archivos_encontrados=$(find "$DIRECTORIO_DOCKING" -type f -path "*/dlg/*" -name
"*${nombre_underscore}*.dlg" 2>/dev/null | head -1)
      fi

      if [[ -n "$archivos_encontrados" && -f "$archivos_encontrados" ]]; then
         if cp "$archivos_encontrados" "$directorio_dlgs" 2>/dev/null; then
            ((archivos_copiados++))
            echo "  Copiado: $(basename "$archivos_encontrados")"
         else
            echo "  Error copiando: $(basename "$archivos_encontrados")"
         fi
      else
         echo "  No encontrado DLG para: $nombre_molecula"

         # Debug: mostrar archivos DLG disponibles
         echo "    DLGs disponibles:"
         find "$DIRECTORIO_DOCKING" -type f -path "*/dlg/*.dlg" -exec basename {} \; 2>/dev/null
| head -5 | while read dlg; do
```

```
            echo "     • $dlg"
          done
        fi
    fi
done < "$archivo_moleculas"

echo "Archivos DLG extraídos: $archivos_copiados archivos copiados"

# Verificar si tenemos DLGs para continuar
if [ $archivos_copiados -eq 0 ]; then
    echo "ADVERTENCIA: No se copiaron archivos DLG. Verificando disponibilidad..."
    find "$DIRECTORIO_DOCKING" -type f -name "*.dlg" | head -10 | while read dlg; do
        echo "  DLG disponible: $(basename "$dlg")"
    done
    return 1
fi


}

function convertir_dlg_a_pdb() { echo "=============================================" echo
"PASO 3: Convirtiendo DLG a PDB..." echo "============================================="

local directorio_dlgs="$DIRECTORIO_RESULTADOS/Dlgs"
local directorio_pdb="$DIRECTORIO_DINAMICA/MejorEnergiaPDB"

mkdir -p "$directorio_pdb"

local archivos_convertidos=0
local archivos_fallidos=0

echo "Extrayendo conformaciones de menor energía..."

# Verificar que MGLTools está configurado correctamente
if [ ! -f "$MGLTOOLS_PYTHON" ]; then
    echo "ERROR: MGLTools Python no encontrado en: $MGLTOOLS_PYTHON"
    return 1
fi

if [ ! -f "$LOWEST_ENERGY_SCRIPT" ]; then
    echo "ERROR: Script write_lowest_energy_ligand.py no encontrado en:
$LOWEST_ENERGY_SCRIPT"
    return 1
fi

for dlg_file in "$directorio_dlgs"/*.dlg; do
    if [[ -f "$dlg_file" ]]; then
        filename=$(basename "$dlg_file" .dlg)
```

```
        output_file="$directorio_pdb/${filename}_lowest_energy.pdb"

        echo "  Procesando: $(basename "$dlg_file")"

        # Cambiar al directorio de destino antes de ejecutar
        cd "$directorio_pdb"
        if run_mgltools_command "$LOWEST_ENERGY_SCRIPT" -f "$dlg_file" -o
"${filename}_lowest_energy.pdb" >/dev/null 2>&1; then
            cd - >/dev/null  # Regresar al directorio anterior
            ((archivos_convertidos++))
            echo "  Convertido: $(basename "$dlg_file")"
        else
            echo "ERROR: Falló la conversión de $(basename "$dlg_file")"
            echo "  Intentando diagnóstico..."

            # Diagnóstico detallado del error
            local temp_log=$(mktemp)
            run_mgltools_command "$LOWEST_ENERGY_SCRIPT" -f "$dlg_file" -o "$output_file" >
"$temp_log" 2>&1

            if [ -s "$temp_log" ]; then
                echo "  Detalles del error:"
                head -5 "$temp_log" | while read line; do
                    echo "    $line"
                done
            fi

            rm -f "$temp_log"
            ((archivos_fallidos++))
        fi
    fi
done

echo "Conversión DLG→PDB completada"
echo "  - Archivos convertidos: $archivos_convertidos"
echo "  - Archivos fallidos: $archivos_fallidos"

if [ "$archivos_fallidos" -gt 0 ]; then
    echo ""
    echo "NOTA: Si persisten los errores de conversión, verifica:"
    echo "  1. Que MGLTools esté instalado correctamente"
    echo "  2. Que los archivos DLG no estén corruptos"
    echo "  3. Que haya suficiente espacio en disco"
fi


}
```

```
function convertir_proteinas_pdb() { echo "============================================"
echo "PASO 4: Convirtiendo proteínas PDBQT a PDB..." echo
"============================================"

local directorio_origen="$DIRECTORIO_RESULTADOS/ProteinaPreparada"
local directorio_destino="$DIRECTORIO_DINAMICA/PDBproteina"

mkdir -p "$directorio_destino"

local archivos_convertidos=0
local archivos_fallidos=0

# Verificar que el script existe
if [ ! -f "$PDBQT_TO_PDB_SCRIPT" ]; then
   echo "ERROR: Script pdbqt_to_pdb.py no encontrado en: $PDBQT_TO_PDB_SCRIPT"
   return 1
fi

for pdbqt_file in "$directorio_origen"/*.pdbqt; do
   if [[ -f "$pdbqt_file" ]]; then
      base_name=$(basename "$pdbqt_file" .pdbqt)
      output_pdb="$directorio_destino/$base_name.pdb"

      echo "  Procesando: $(basename "$pdbqt_file")"

      # Usar la función run_mgltools_command que configura el entorno correctamente
      if run_mgltools_command "$PDBQT_TO_PDB_SCRIPT" -f "$pdbqt_file" -o "$output_pdb"
>/dev/null 2>&1; then
         ((archivos_convertidos++))
         echo "  ✓ Convertido: $(basename "$pdbqt_file")"
      else
         echo "ERROR: No se pudo convertir $(basename "$pdbqt_file")"

         # Diagnóstico del error
         local temp_log=$(mktemp)
         run_mgltools_command "$PDBQT_TO_PDB_SCRIPT" -f "$pdbqt_file" -o "$output_pdb" >
"$temp_log" 2>&1

         if [ -s "$temp_log" ]; then
            echo "  Detalles del error:"
            head -3 "$temp_log" | while read line; do
               echo "    $line"
            done
         fi

         rm -f "$temp_log"
         ((archivos_fallidos++))
      fi
```

```
    fi
done

echo "✓ Conversión proteínas completada"
echo "  - Archivos convertidos: $archivos_convertidos"
echo "  - Archivos fallidos: $archivos_fallidos"


}

function reparar_ligandos() { echo "==============================================" echo
"PASO 5: Reparando ligandos para dinámicas..." echo
"=============================================="

local directorio_ligandos_originales="$DIRECTORIO_DINAMICA/MejorEnergiaPDB"
local directorio_ligandos_reparados="$DIRECTORIO_DINAMICA/LigandosReparados"
local
script_reparacion="$MGLTOOLS_PATH/MGLToolsPckgs/AutoDockTools/Utilities24/repair_ligand
4.py"
local
prepare_ligand_script="$MGLTOOLS_PATH/MGLToolsPckgs/AutoDockTools/Utilities24/prepare_
ligand4.py"

mkdir -p "$directorio_ligandos_reparados"

# Verificar que existen los scripts necesarios
if [[ ! -f "$script_reparacion" ]]; then
    echo "ERROR: Script de reparación no encontrado: $script_reparacion"
    return 1
fi

if [[ ! -f "$prepare_ligand_script" ]]; then
    echo "ERROR: Script prepare_ligand4.py no encontrado: $prepare_ligand_script"
    return 1
fi

local ligandos_reparados=0
local ligandos_fallidos=0

echo "Aplicando reparaciones estructurales a los ligandos..."

# Convertir PDB a PDBQT para reparación
for pdb_file in "$directorio_ligandos_originales"/*.pdb; do
    if [[ -f "$pdb_file" ]]; then
        base_name=$(basename "$pdb_file" .pdb)
        pdbqt_temp="$directorio_ligandos_reparados/${base_name}_temp.pdbqt"
        pdbqt_reparado="$directorio_ligandos_reparados/${base_name}_repaired.pdbqt"
        pdb_final="$directorio_ligandos_reparados/${base_name}_repaired.pdb"
```

```
        echo "  Procesando: $base_name"

        # Paso 1: Convertir PDB a PDBQT temporal usando prepare_ligand4.py
        local temp_log=$(mktemp)
        if run_mgltools_command "$prepare_ligand_script" -l "$pdb_file" -o "$pdbqt_temp" >
"$temp_log" 2>&1; then

            # Paso 2: Aplicar reparación con repair_ligand4.py
            if run_mgltools_command "$script_reparacion" -f "$pdbqt_temp" -o "$pdbqt_reparado"
>/dev/null 2>&1; then

                # Paso 3: Convertir PDBQT reparado de vuelta a PDB
                if run_mgltools_command "$PDBQT_TO_PDB_SCRIPT" -f "$pdbqt_reparado" -o
"$pdb_final" >/dev/null 2>&1; then
                    ((ligandos_reparados++))
                    echo "    ✓ Reparado exitosamente"

                    # Limpiar archivos temporales
                    rm -f "$pdbqt_temp" "$pdbqt_reparado" 2>/dev/null
                else
                    echo "    ✗ Error en conversión final a PDB"
                    ((ligandos_fallidos++))
                fi
            else
                echo "    ✗ Error en reparación estructural"
                ((ligandos_fallidos++))
            fi
        else
            echo "    ✗ Error: prepare_ligand4.py falló"
            # Diagnóstico silenciado - continuar con método alternativo

            # Intentar método alternativo: usar el ligando original sin reparar
            echo "    → Intentando usar ligando original sin reparación..."
            if cp "$pdb_file" "$directorio_ligandos_reparados/${base_name}_repaired.pdb" 2>/dev/null;
then
                echo "    ✓ Copiado como ligando sin reparar"
                ((ligandos_reparados++))
            else
                echo "    ✗ Error al copiar ligando original"
                ((ligandos_fallidos++))
            fi
        fi
        rm -f "$temp_log"
    fi

    # Limpiar archivos temporales si quedan
    rm -f "$pdbqt_temp" "$pdbqt_reparado" 2>/dev/null
```

```
done

echo "✓ Reparación de ligandos completada"
echo "  - Ligandos reparados exitosamente: $ligandos_reparados"
echo "  - Ligandos con errores: $ligandos_fallidos"

# Verificar si tenemos ligandos reparados para continuar
if [ $ligandos_reparados -eq 0 ]; then
    echo "ADVERTENCIA: No se repararon ligandos. Usando ligandos originales para complejos."
    return 1
fi


}

function crear_complejos_moleculares() { echo
"==============================================" echo "PASO 6: Creando complejos
moleculares..." echo "=============================================="

local directorio_ligandos_reparados="$DIRECTORIO_DINAMICA/LigandosReparados"
local directorio_ligandos_originales="$DIRECTORIO_DINAMICA/MejorEnergiaPDB"
local directorio_proteinas="$DIRECTORIO_DINAMICA/PDBproteina"
local directorio_complejos="$DIRECTORIO_DINAMICA/Complex"

mkdir -p "$directorio_complejos"

# Determinar qué directorio de ligandos usar
local directorio_ligandos
if [ -d "$directorio_ligandos_reparados" ] && [ "$(find "$directorio_ligandos_reparados" -name
"*.pdb" | wc -l)" -gt 0 ]; then
    directorio_ligandos="$directorio_ligandos_reparados"
    echo "Usando ligandos reparados para crear complejos..."
else
    directorio_ligandos="$directorio_ligandos_originales"
    echo "Usando ligandos originales (reparación no disponible)..."
fi

# Buscar archivo de proteína (debe ser único)
local archivo_proteina=$(find "$directorio_proteinas" -type f -name "*.pdb" | head -n 1)

if [ -z "$archivo_proteina" ]; then
    echo "ERROR: No se encontró archivo de proteína en $directorio_proteinas"
    return 1
fi

echo "Usando proteína: $(basename "$archivo_proteina")"

local complejos_creados=0
```

```
# Crear complejo para cada ligando
for ligando in "$directorio_ligandos"/*.pdb; do
  if [ -f "$ligando" ]; then
    base_name=$(basename "$ligando" .pdb)
    # Limpiar el nombre para crear una carpeta válida
    carpeta_nombre=$(echo "$base_name" | sed 's/[^a-zA-Z0-9_-]/_/g')
    carpeta_complejo="$directorio_complejos/$carpeta_nombre"

    mkdir -p "$carpeta_complejo"

    # Copiar ligando y proteína a la carpeta del complejo
    if cp "$ligando" "$carpeta_complejo/" && cp "$archivo_proteina" "$carpeta_complejo/"; then

      # Crear archivo de complejo combinado
      archivo_complejo="$carpeta_complejo/complejo.pdb"

      # Combinar archivos: ligando + proteína
      grep -v '^END' "$ligando" > "$archivo_complejo"
      grep -v '^HEADER\|^TITLE\|^END' "$archivo_proteina" >> "$archivo_complejo"
      echo "END" >> "$archivo_complejo"

      ((complejos_creados++))
      echo "  ✓ Complejo creado: $carpeta_nombre"
    else
      echo "ERROR: No se pudo crear complejo para $(basename "$ligando")"
    fi
  fi
done

echo "✓ Complejos moleculares creados: $complejos_creados"


}

function organizar_resultados_finales() { echo
"==========================================" echo "PASO 7: Organizando estructura
final..." echo "=========================================="

# Crear estructura de directorios final
mkdir -p "$DIRECTORIO_DINAMICA/DockingResults"
mkdir -p "$DIRECTORIO_ARCHIVOS_DOCKING"

# Mover archivos a estructura final
echo "Reorganizando archivos..."

# Mover archivos de docking a DockingResults
[ -d "$DIRECTORIO_RESULTADOS/Dlgs" ] && mv "$DIRECTORIO_RESULTADOS/Dlgs"
```

```
"$DIRECTORIO_DINAMICA/DockingResults/" 2>/dev/null
[ -f "$DIRECTORIO_RESULTADOS/nombres_moleculas.txt" ] && mv
"$DIRECTORIO_RESULTADOS/nombres_moleculas.txt"
"$DIRECTORIO_DINAMICA/DockingResults/" 2>/dev/null

# Mover carpetas de preparación a ArchivosDocking
[ -d "$DIRECTORIO_RESULTADOS/MoleculaPreparada" ] && mv
"$DIRECTORIO_RESULTADOS/MoleculaPreparada" "$DIRECTORIO_ARCHIVOS_DOCKING/"
2>/dev/null
[ -d "$DIRECTORIO_RESULTADOS/MoleculaOptimizada" ] && mv
"$DIRECTORIO_RESULTADOS/MoleculaOptimizada" "$DIRECTORIO_ARCHIVOS_DOCKING/"
2>/dev/null
[ -d "$DIRECTORIO_RESULTADOS/ProteinaPreparada" ] && mv
"$DIRECTORIO_RESULTADOS/ProteinaPreparada" "$DIRECTORIO_ARCHIVOS_DOCKING/"
2>/dev/null

echo "✓ Estructura de resultados organizada"


}

function generar_reporte_final() { echo "============================================" echo
"PASO 8: Generando reporte final..." echo "============================================"

local archivo_reporte="$DIRECTORIO_RESULTADOS/Reporte_Analisis.txt"

{
   echo "============================================"
   echo "REPORTE DE ANÁLISIS DE DOCKING MOLECULAR"
   echo "SDASAM 3.0 - $(date)"
   echo "============================================"
   echo ""

   # Estadísticas generales
   echo "ESTADÍSTICAS GENERALES:"
   echo "----------------------"

   if [ -f "$DIRECTORIO_DINAMICA/DockingResults/nombres_moleculas.txt" ]; then
      num_moleculas=$(wc -l <
"$DIRECTORIO_DINAMICA/DockingResults/nombres_moleculas.txt")
      echo "• Número de moléculas procesadas: $num_moleculas"
   fi

   if [ -d "$DIRECTORIO_DINAMICA/DockingResults/Dlgs" ]; then
      num_dlgs=$(find "$DIRECTORIO_DINAMICA/DockingResults/Dlgs" -name "*.dlg" | wc -l)
      echo "• Archivos DLG extraídos: $num_dlgs"
   fi
```

```
    if [ -d "$DIRECTORIO_DINAMICA/LigandosReparados" ]; then
        num_reparados=$(find "$DIRECTORIO_DINAMICA/LigandosReparados" -name "*.pdb" | wc -l)
        echo "• Ligandos reparados estructuralmente: $num_reparados"
    fi

    if [ -d "$DIRECTORIO_DINAMICA/MejorEnergiaPDB" ]; then
        num_ligandos=$(find "$DIRECTORIO_DINAMICA/MejorEnergiaPDB" -name "*.pdb" | wc -l)
        echo "• Ligandos con menor energía extraídos: $num_ligandos"
    fi

    if [ -d "$DIRECTORIO_DINAMICA/Complex" ]; then
        num_complejos=$(find "$DIRECTORIO_DINAMICA/Complex" -mindepth 1 -type d | wc -l)
        echo "• Complejos moleculares creados: $num_complejos"
    fi

    echo ""
    echo "NOMBRES DE MOLÉCULAS PROCESADAS:"
    echo "-------------------------------"
    if [ -f "$DIRECTORIO_DINAMICA/DockingResults/nombres_moleculas.txt" ]; then
        while IFS= read -r nombre; do
            echo "• $nombre"
        done < "$DIRECTORIO_DINAMICA/DockingResults/nombres_moleculas.txt"
    fi

    echo ""
    echo "ESTRUCTURA DE RESULTADOS:"
    echo "------------------------"
    echo "• $DIRECTORIO_DINAMICA/DockingResults/ - Archivos de docking procesados"
    echo "• $DIRECTORIO_DINAMICA/MejorEnergiaPDB/ - Conformaciones de menor energía"
    echo "• $DIRECTORIO_DINAMICA/LigandosReparados/ - Ligandos con reparaciones estructurales"
    echo "• $DIRECTORIO_DINAMICA/PDBproteina/ - Proteínas en formato PDB"
    echo "• $DIRECTORIO_DINAMICA/Complex/ - Complejos listos para dinámicas"
    echo "• $DIRECTORIO_ARCHIVOS_DOCKING/ - Archivos de preparación"
    echo ""

    echo "Los resultados están listos para análisis de dinámica molecular."
    echo "=================================================="

} > "$archivo_reporte"

echo "✓ Reporte generado: $archivo_reporte"


}
```

```
function main() { echo "Iniciando análisis completo de resultados..." echo "Directorio base:
$DIRECTORIO_BASE" echo ""

# Verificar directorio base
if [ ! -d "$DIRECTORIO_RESULTADOS" ]; then
    echo "ERROR: Directorio de resultados no encontrado: $DIRECTORIO_RESULTADOS"
    exit 1
fi

# Verificar MGLTools antes de comenzar
if [ ! -f "$MGLTOOLS_PYTHON" ]; then
    echo "ERROR: MGLTools Python no encontrado en: $MGLTOOLS_PYTHON"
    echo "Verifica la instalación de MGLTools"
    exit 1
fi

if [ ! -f "$LOWEST_ENERGY_SCRIPT" ]; then
    echo "ERROR: Script write_lowest_energy_ligand.py no encontrado en:
$LOWEST_ENERGY_SCRIPT"
    echo "Verifica la instalación completa de AutoDockTools"
    exit 1
fi

echo "✓ Verificación de dependencias completada"
echo "  - MGLTools Python: $(basename "$MGLTOOLS_PYTHON")"
echo "  - Scripts de conversión: disponibles"
echo ""

# Ejecutar todas las funciones en secuencia
analizar_datos_docking || { echo "ERROR en análisis de datos"; exit 1; }
extraer_archivos_dlg || { echo "ERROR en extracción DLG"; exit 1; }
convertir_dlg_a_pdb || { echo "ERROR en conversión DLG→PDB"; exit 1; }
convertir_proteinas_pdb || { echo "ERROR en conversión proteínas"; exit 1; }

# Intentar reparar ligandos (opcional, no crítico)
echo "Intentando reparar ligandos..."
if reparar_ligandos; then
    echo "✓ Reparación de ligandos completada exitosamente"
else
    echo "⚠ Reparación de ligandos falló o no fue necesaria. Continuando con ligandos originales..."
fi

crear_complejos_moleculares || { echo "ERROR en creación de complejos"; exit 1; }
organizar_resultados_finales
generar_reporte_final

echo ""
echo "=========================================="
```

```
echo "    ANÁLISIS COMPLETO FINALIZADO          "
echo "========================================"
echo "Todos los resultados han sido procesados y organizados."
echo "Los complejos están listos para dinámicas moleculares."
echo "Consulte el reporte en: $DIRECTORIO_RESULTADOS/Reporte_Analisis.txt"
echo ""
echo "NOTA: Si experimentaste errores con MGLTools, verifica:"
echo "  1. Instalación completa de MGLTools en: $MGLTOOLS_PATH"
echo "  2. Presencia de AutoDockTools en MGLToolsPckgs/"
echo "  3. Permisos de lectura en los directorios de MGLTools"


}

if [[ "${BASH_SOURCE[0]}" == "${0}" ]]; then main "$@" fi
```
